# Decoupled supercapacitive electrolyzer for membrane-free water splitting

**DOI:** 10.1126/sciadv.adi3180

**Published:** 2024-03-06

**Authors:** Esteban A. Toledo-Carrillo, Mario García-Rodríguez, Lorena M. Sánchez-Moren, Joydeep Dutta

**Affiliations:** ^1^ Functional NanoMaterials Group, Department of Applied Physics, School of Engineering Sciences, KTH Royal Institute of Technology, Hannes Alfvéns väg 12, 114 19 Stockholm, Sweden.; ^2^ Departamento de Química Física e Instituto Universitario de Materiales, Universidad de Alicante, Ap. 99, E-03080, Alicante, Spain.; ^3^ Departamento de Química Inorgánica e Instituto Universitario de Materiales, Universidad de Alicante, Ap. 99, E-03080, Alicante, Spain.

## Abstract

Green hydrogen production via water splitting is vital for decarbonization of hard-to-abate industries. Its integration with renewable energy sources remains to be a challenge, due to the susceptibility to hazardous gas mixture during electrolysis. Here, we report a hybrid membrane-free cell based on earth-abundant materials for decoupled hydrogen production in either acidic or alkaline medium. The design combines the electrocatalytic reactions of an electrolyzer with a capacitive storage mechanism, leading to spatial/temporal separation of hydrogen and oxygen gases. An energy efficiency of 69% lower heating value (48 kWh/kg) at 10 mA/cm^2^ (5 cm–by–5 cm cell) was achieved using cobalt-iron phosphide bifunctional catalyst with 99% faradaic efficiency at 100 mA/cm^2^. Stable operation over 20 hours in alkaline medium shows no apparent electrode degradation. Moreover, the cell voltage breakdown reveals that substantial improvements can be achieved by tunning the activity of the bifunctional catalyst and improving the electrodes conductivity. The cell design offers increased flexibility and robustness for hydrogen production.

## Teaser

Spatial gas separation is enabled by capacitive storage during water splitting in membraneless cell design.

## INTRODUCTION

The urgent need to decarbonize the economy has promoted the pursuit for energy generation from renewable sources and the use of clean fuels (*1*). Hydrogen is considered to be a promising candidate to reduce the use of fossil fuels in hard-to-abate industries, due to its high energy density and the possible usage as a feedstock for chemical production or as energy carrier to feed fuel cells or combustion engines producing only water as by-product (*2*–*5*). Now, hydrogen is produced mainly by steam methane reforming, accounting for more than 95% of global production and ~3% of global CO_2_ emissions (*6*, *7*). Hydrogen can be produced by water splitting via different methods such as thermochemical processes (*8*), photocatalysis (*9*, *10*), photoelectrochemical (*11*, *12*), and electrolysis (*13*, *14*). The need for decarbonization strategies coupled with the rapid commercial implementation of renewable energy generation systems has led to considerable attention in hydrogen production via water electrolysis because it allows the production of green hydrogen using only water and clean energy as input, with practically zero CO_2_ emissions. There are four leading water electrolysis technologies deployed worldwide, namely, alkaline water electrolyzer, proton exchange membrane electrolyzer (PEM), anion exchange membrane electrolyzer (AEM), and solid oxide electrolyzer (SOE). Alkaline electrolysis proposed by Troostwijk and Diemann in 1789 is today a mature technology (*15*) that is widely implemented in industrial processes with the advantage of using earth-abundant catalysts, but it operates at low current densities (~400 to 800 mA/cm^2^). PEM electrolyzers in retrospect offer higher production rate and compact design but require the use of platinum group metals (PGMs) and membranes that can withstand acidic conditions. AEM combines the benefits of the use of membranes and the mild corrosivity of alkaline electrolytes. SOE has the potential to operate at higher energy efficiencies, but it requires high temperature and is still in its early stage of development.

Large-scale implementation of water electrolysis systems requires efficient and reliable stacks, and some of the ongoing challenges are yet to be addressed in conventional electrolyzers. The use of renewable sources and the intermittent nature of the energy generation requires operation at partial load that is still a major issue in conventional electrolyzers due to the crossover of gases across the membrane, requiring additional purification steps (*16*). Uncontrolled gas diffusion can also result in explosive mixtures of H_2_ and O_2_ that is a major challenge for the safety of a system, and, thus, the generated pressures of H_2_ and O_2_ must be carefully controlled to prevent permeation between the anodic and cathodic compartments.

After over two centuries of coupled water electrolysis, decoupled water splitting is a paradigm shift from simultaneous hydrogen and oxygen evolution offering highly efficient, low-cost, and robust hydrogen production process. The concept of decoupling hydrogen production was introduced to separate the generation of H_2_ and O_2_ in time or space (*17*–*23*). Symes and Cronin (*24*) developed a two-step electrochemical system where a first step consists of water oxidation, while electrons and proton react with polyoxometalate (electron-coupled proton buffer) to reoxidize it during the second step, providing the electrons and protons to activate hydrogen evolution reaction (HER). Other redox mediators such as V(II)/V(III), Ce(III)/Ce(IV), H_4_[SiW_12_O_40_] (silicotungstic acid), sodium ferrocyanide (Na_4_[Fe(CN)_6_]). and hydroquinone sulfonate (C_6_H_5_O_5_S^−^) have also been proposed (*25*–*31*). Most of these designs require membranes to separate the redox mediators from the electrocatalyst, and, thus, solid-state mediators have been introduced to build membrane-free electrolyzer using battery-like materials (*32*–*35*). However, redox materials suffer from poor reversibility and substantial capacity losses at high current densities, leading to higher energy requirements for sustained water electrolysis.

In this work, we have extended the concept of supercapacitive electrolyzer (SCE) for decoupled hydrogen production developed by our group in 2021 (*36*, *37*) that has recently been independently validated by Guo *et al.* (*38*)*.* The SCE introduces a simpler working concept of decoupled water splitting as it is a membrane-free hybrid cell architecture combining the standard electrocatalytic reactions of an electrolyzer to a capacitive storage mechanism. We have explored the application of the cell design in both acidic and alkaline conditions using standard platinum electrode (Pt/C). It was further optimized using the bimetallic phosphide bifunctional catalyst (CoFeP) to demonstrate the influence of the overall water splitting activity as well as to show the limitations and challenges for the use of bifunctional catalysts. Overall, an energy consumption of 48 kWh/kg was obtained in alkaline conditions, which can be further improved by replacing activated carbon cloth (ACC) with a highly conductive auxiliary electrode.

## RESULTS

### Concept validation

The production of hydrogen was envisioned in an electrochemical hybrid system, combining the standard electrocatalytic reactions of an electrolyzer together with a capacitive storage mechanism to decouple hydrogen generation from oxygen generation either spatially or over time. The SCE system is composed of two identical cells to maintain a continuous production of hydrogen. The detailed construction of each cell is schematically represented in fig. S1.

**Fig. 1.**
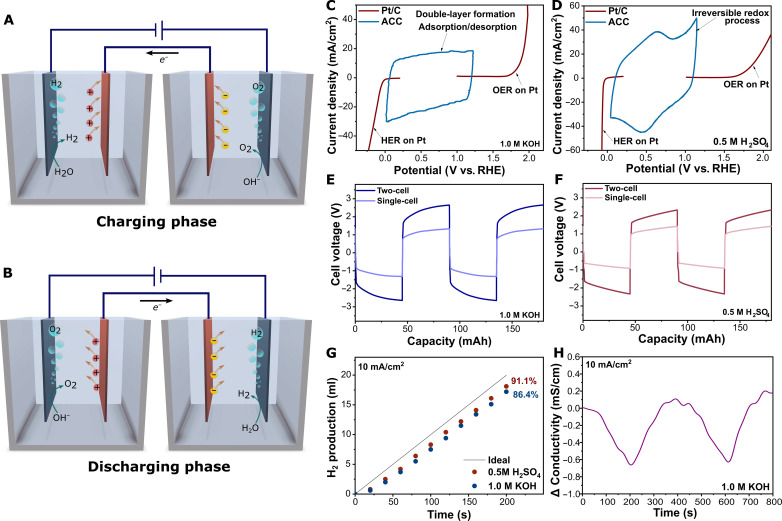
SCE design and proof of concept. (**A**) Proposed mechanism in the anodic half-cell and the cathodic half-cell during charging step and (**B**) discharging step. Voltammogram of ACC and Pt/C obtained with a scan rate of 5 mV/s in (**C**) 1.0 M KOH and (**D**) 0.5 M H_2_SO_4_. Voltage profile considering single-cell and two-cell contribution in (**E**) 1.0 M KOH and (**F**) 0.5 M H_2_SO_4._ (**G**) Hydrogen production in both alkaline and acidic media. (**H**) Conductivity in the alkaline reservoir for two consecutive cycles.

The working principle of the two-step process is illustrated in Fig. 1 (A and B). The process involves two distinct steps, namely, charging and discharging, which are named after the charging state of the auxiliary electrode. During the charging step, the bifunctional electrode placed in the cathodic cell promotes HER, leading to the production of hydrogen exclusively from the cathodic cell. Simultaneously, the bifunctional electrode in the anodic cell promotes the oxygen evolution reaction (OER) to generate oxygen exclusively in the anodic cell. This process occurs in separate chambers, allowing the effective separation of the gases without the need of a membrane. The process is mediated by the charging of the auxiliary electrodes placed in each cell, through the formation of an electrochemical double layer analogous to the storage mechanism of supercapacitors. In the cathodic cell, the capacitive electrode adsorbs OH^−^ ions, while, in the anodic cell, K^+^ ions are adsorbed when KOH is the electrolyte (alkaline cell), as illustrated in Fig. 1A.

To evaluate this concept of capacitive-mediated electrolysis and validate the hypothesis, we constructed a system comprising a conventional HER catalyst, such as Pt/C, with commercial ACC as auxiliary electrode. Detailed characterization of Pt/C is presented in the Supplementary Materials (figs. S2 and S3). The system was evaluated with both acidic and alkaline electrolytes to demonstrate its operational flexibility. The catalytic and capacitive properties of the electrodes were studied by linear sweep voltammetry (LSV) and cyclic voltammetry (CV), respectively. LSV of Pt/C reveals its superior electrocatalytic activity toward HER in both acidic and alkaline media, with overpotentials of 55 and 77 mV, respectively, at a current density of 10 mA/cm^2^ (Fig. 1, C and D). In contrast, Pt/C demonstrates a poor activity toward OER, with faster kinetics observed in alkaline medium at higher current densities. The overpotentials required to reach a current density of 10 mA/cm^2^ are 643 and 616 mV in alkaline and acidic media, respectively. The capacitive storage of ACC is demonstrated by the quasi-rectangular shape of the cyclic voltammogram in alkaline media shown in Fig. 1C, whereas, in acidic medium (Fig. 1D), a reversible redox pair is observed at 0.5 V versus reversible hydrogen electrode (RHE), which corresponds to the reversible oxidation processes of the ACC-oxygenated groups (*39*).

The cell voltage of the system (two-cell) indicating the contribution of the cell is shown in Fig. 1 (E and F). Two distinguishable features are clearly visible in the voltage profile. The steady increase in cell voltage is associated to the buildup potential on the capacitive electrode during galvanostatic charging, while the initial voltage is dependent on the catalyst and the series resistance of the cell. To further validate the cell design, the conductivity of the electrolytes was monitored over time to identify changes in the concentration of the solution (Fig. 1H). As expected, a cyclic pattern is observed in the conductivity profile, showing a reduction in conductivity during charging step and subsequent increase up to the initial level, during the discharging step, further validating the occurrence of the capacitive storage mechanism mediated by adsorption/desorption process. Gas evolution from the cathodic cell during decoupled electrolysis in both acidic and alkaline media is shown in Fig. 1G. Faradaic efficiency for hydrogen production is slightly higher in acidic medium of 91.1%, compared to 86.4% in alkaline medium. Losses arise during the first 10 s of applied current, and the production rate after that reaches a faradaic efficiency of 99%.

Thus, the concept of SCE proposed in this study has been demonstrated to be effective in decoupling the two catalytic reactions of water splitting in space and is able to operate in a wide range of pH. However, the cell voltage requirement of the system based on Pt/C is high due to the low activity of Pt toward OER. The energy consumption and energy efficiency are highly dependent on the activity of the electrocatalyst.

### Bifunctional and auxiliary electrodes

**Fig. 2.**
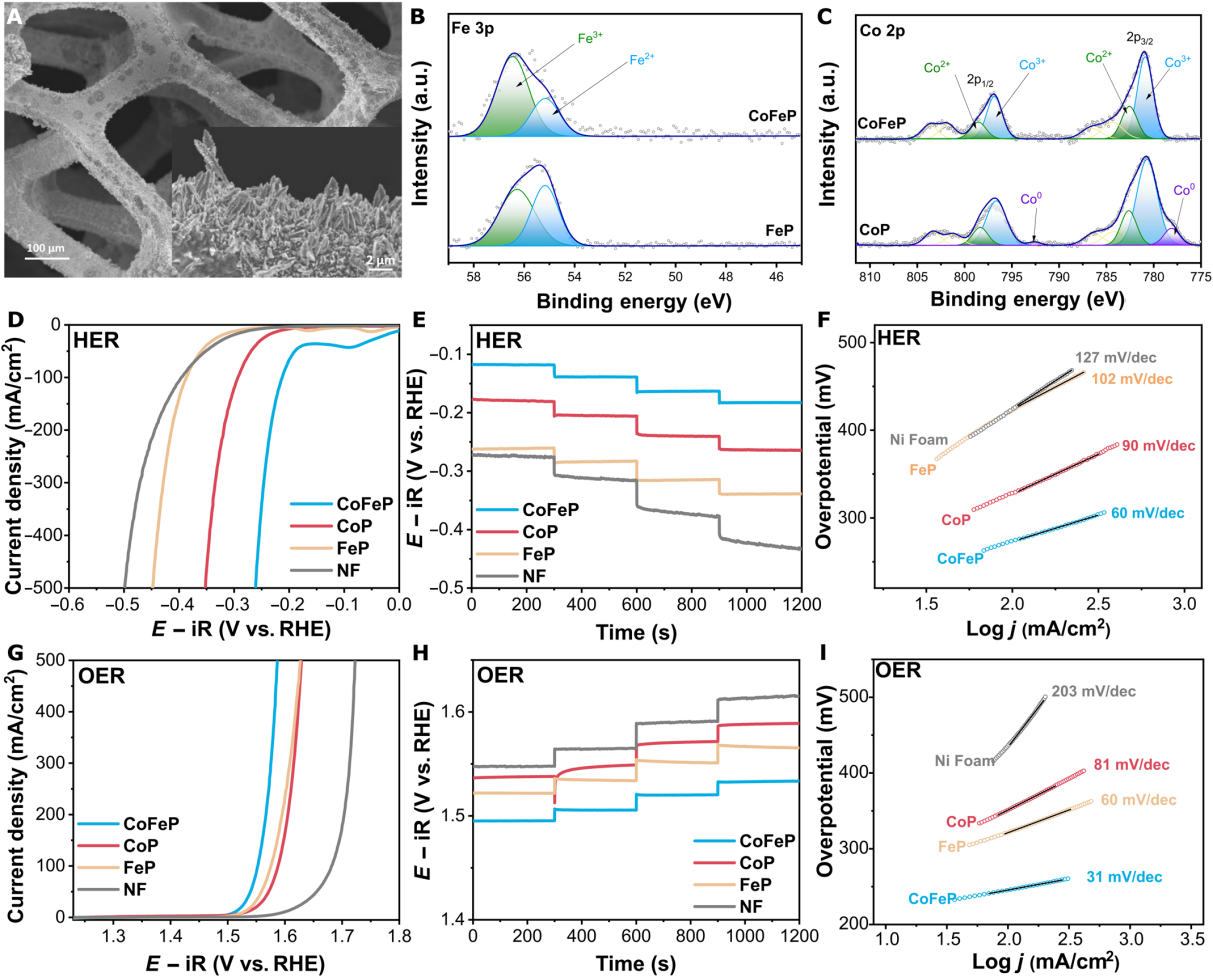
Characterization of bifunctional electrocatalyst. (**A**) Scanning electron microscope image of CoFeP deposited on Ni Foam (NF). Catalysts high-resolution core-level x-ray photoelectron spectroscopy (XPS) spectra of (**B**) Fe 3p and (**C**) Co 2p. a.u., arbitrary units. (**D**) Cathodic LSV at a scan rate of 5 mV/s, (**E**) chronopotentiometry tests at different cathodic current densities, and (**F**) Tafel slopes of electrocatalysts from cathodic polarization in 1.0 M KOH. (**G**) Anodic LSV at a scan rate of 5 mV/s, (**H**) chronopotentiometry tests at different anodic current densities, and (**I**) Tafel slopes of electrocatalysts from anodic polarization in 1.0 M KOH.

To further demonstrate the promising decoupled electrolysis system proposed herein, a highly active electrocatalyst toward both the HER and OER is needed. Among the wide variety of bifunctional catalysts reported in literature (carbides, nitrides, sulfide, oxides, hydroxides, alloys, metal organic frameworks (MOFs), and molecular catalysts) (*40*), transition metal–based phosphides are regarded as a promising electrocatalyst due to its high activity and stability in alkaline conditions. Cobalt-iron phosphide (CoFeP) bifunctional catalyst was thus prepared by a one-step galvanostatic electrodeposition method on Ni foam (fig. S4). The synthesis was performed in a three-electrode setup as described in fig. S5. The electrodeposition of single and bimetallic phosphides was confirmed by scanning electron microscope, as shown in Fig. 2A and figs. S6 and S7. A uniform and dense layer of ~1-μm thickness was obtained for CoFeP with appreciable dendritic growth due to the high current density applied during electrodeposition (inset of Fig. 2A and fig. S8). Comparatively, CoP shows less dendritic growth and smoother surface (fig. S6). The dendritic structure onto the porous structure of Ni Foam provides a facile access to active sites, efficient removal of the gaseous products in a flow through architecture as well as providing a three-dimensional (3D) conductive framework and sufficient surface area for the catalytic processes to take place (*41*).

The chemical composition of CoFeP was studied by inductively coupled plasma optical emission spectroscopy (ICP-OES) and x-ray photoelectron spectroscopy (XPS). The metal content was quantified by ICP-OES, obtaining a ratio Fe/Co of 0.4. The XPS survey spectrum confirms the presence of Co, Fe, P, O, and Ni in CoFeP sample (Fig. 2, B and C, and fig. S9). High-resolution Co 2p spectra of the CoP and CoFeP samples are shown in Fig. 2C. Two distinctive regions located could be observed at ~781.1 and 797.0 eV, corresponding to the 2p_3/2_ and 2p_1/2_ regions. The 2p_3/2_ region can be further deconvulated into peaks at 778.0, 780.7, 782.7, 784.3, and 786.4 eV, which are associated with Co^0^, Co^3+^, and Co^2+^ species, with two satellite peaks, respectively. Similarly, the 2p_1/2_ region can be deconvulated into peaks at 792.7, 796.6, 798.3, 801.1, and 803.3 eV, which are related to Co^0^, Co^3+^, and Co^2+^ species, and two satellite peaks, respectively. Moreover, the Fe 3p region is displayed in Fig. 2B, with a primary peak at ~56 eV for FeP and CoFeP samples. The 3p peak can be resolved into two contributions at 56.3 and 55.1 eV, which are ascribed to Fe^3+^ and Fe^2+^, respectively. Co has a higher redox potential and forms a Co─Fe bond with a net transfer of electron from Fe to Co leading to a change in the oxidation state of Fe, resulting in the formation of Fe^3+^ (*42*). This interaction influences the electronic structure of the compound which can be seen also in the O 1s spectra (fig. S9B) with appreciable shift to higher binding energies in the FeCoP sample due to the more electronegative nature of the Fe-Co interaction.

The electrochemical activity was first evaluated toward HER by means of LSV in 1.0 M KOH (Fig. 2D). It can be observed that bare Ni foam has a poor activity toward HER with a large onset potential. In fig. S10, we have summarized the overpotentials of all the catalysts studied in this work. CoFeP showed an overpotential of 205, 251, and 273 mV at current densities of 10, 50, and 100 mA/cm^2^, respectively; whereas CoP and FeP show an overpotential of 329 and 423 mV to reach a current density of 100 mA/cm^2^. The Tafel slope for Ni Foam was estimated to be 126 mV/decade (dec), close to reported values in alkaline medium, indicating a Volmer-limited reaction (*43*, *44*). Improved performance is observed for both single-metal FeP and CoP, and CoFeP with Tafel slopes of 102, 90, and 60 mV/dec, respectively (Fig. 2F), indicating a Volmer-Heyrovsky reaction mechanism of the latter (*45*). Improved HER kinetics of CoFeP has been attributed to the modulation of the electronic structure around Co active sites by Fe atoms, as discussed above during the analysis of the results obtained from XPS. It has been reported that this phenomenon leads to a lower adsorption energy of hydrogen intermediates (*46*, *47*). The activity of CoFeP was further evaluated by chronopotentiometry as shown in Fig. 2E. Current density was ramped up from 10 to 100 mA/cm^2^ to assess the response of the electrode under dynamic external power. With CoFeP electrodes, a steady potential was reached within 5 to 10 s of the application of power, similar to the control samples.

Similarly, the OER performance of all samples was studied in alkaline conditions. LSV curve shows a clear improvement in activity of CoFeP compared to the single-metal phosphides (Fig. 2G). An overpotential of 210, 237, and 245 mV was required to achieve current densities of 10, 50, and 100 mA/cm^2^, respectively (fig. S10A). Tafel slope is notably reduced from 60 mV/dec (FeP) and 81 mV/dec (CoP) to 31 mV/dec for CoFeP, suggesting a modulation in the adsorption energy because the energy barrier for the rate determining step of the reaction is reduced (Fig. 2I). Chronopotentiometric study (Fig. 2H) revealed a fast response to changes in current densities during OER, whereas the control samples show a slow and continuous change in potential. The electrochemically active surface area (ECSA) was investigated to determine its role in electrocatalytic activity. The ECSA was estimated from the capacitance of the double layer (*C*_dl_) and plotted in fig. S11. The *C*_dl_ of CoFeP (26.6 mF/cm^2^) shows a 10-fold increase compared to the Ni substrates (2.4 mF/cm^2^), associated to the dendritic growth and is comparable to the active surface area obtained for similar catalyst structures. Single-metal catalysts in retrospect show a modest increase in active surface area.

LSV was carried out in a two-electrode setup to study the overall water splitting performance of the bifunctional catalyst, and the results are plotted fig. S12. The cell voltage necessary to obtain a current density of 50 mA/cm^2^ was 1.76 V versus RHE, indicating an important reduction in overall water splitting potential upon using CoFeP in comparison to the Pt/C electrodes as discussed above.

The stability of CoFeP bifunctional catalysts was studied using galvanostatic charging-discharging cycles of 400 s at 20 mA/cm^2^. No evident degradation of electrode potential profile could be observed over 16 hours of stability test at 20 mA/cm^2^ as a constant potential for both HER and OER reactions were registered (fig. S13). It is noteworthy to state that the HER and OER potential are reached within 5 to 10 s after switching, which indicates that losses due to parasitic redox reactions are limited.

**Fig. 3.**
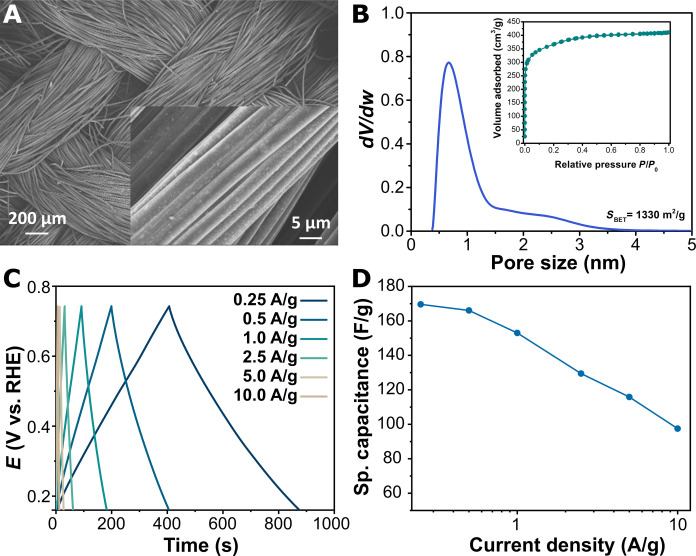
Characterization of capacitive electrode. (**A**) Scanning electron microscope image of ACC. (**B**) Pore size distribution; inset: N_2_ adsorption isotherm. (**C**) Galvanostatic charge discharge profile at different current densities in 1.0 M KOH. (**D**) Specific capacitance obtained from the galvanostatic charge-discharge (GCD) test.

The auxiliary electrode made of ACC was examined by scanning microscopy, as shown in Fig. 3A. The electrode consists of carbon fibers of 5 to 10 μm with an apparent smooth surface. XPS characterization can be seen in fig. S14. N_2_ adsorption measurements yield a surface area of 1330 m^2^/g as estimated from Brunauer-Emmett-Teller (BET) method with a pore size distribution predominantly in the range of 0.4 to 3 nm (micropores) as shown in Fig. 3B. Capacitive storage capabilities of ACC were studied by CV and galvanostatic charge-discharge (GCD) in the potential windows of water stability to simulate operating conditions in the SCE. Figure S15 shows the voltammogram of ACC at different scan rates, clearly showing the quasi-rectangular shape in the potential windows between 0.05 V and 1.2 V versus RHE, associated to capacitive storage. The charge storage was evaluated by GCD at current densities up to 10 A/g (Fig. 3C) because fast charging will be crucial for the application of the electrode material in an SCE. The potential window considered during GCD was between 0.15 and 0.75 V versus RHE to limit the oxidation of carbon electrode during long-term operation. The specific capacitance calculated from GCD was 169 F/g at a charging rate of 0.25 A/g and further reduced to 97.5 F/g during charging at 10 A/g (Fig. 3D).

### Hydrogen production in a single-cell SCE

**Fig. 4.**
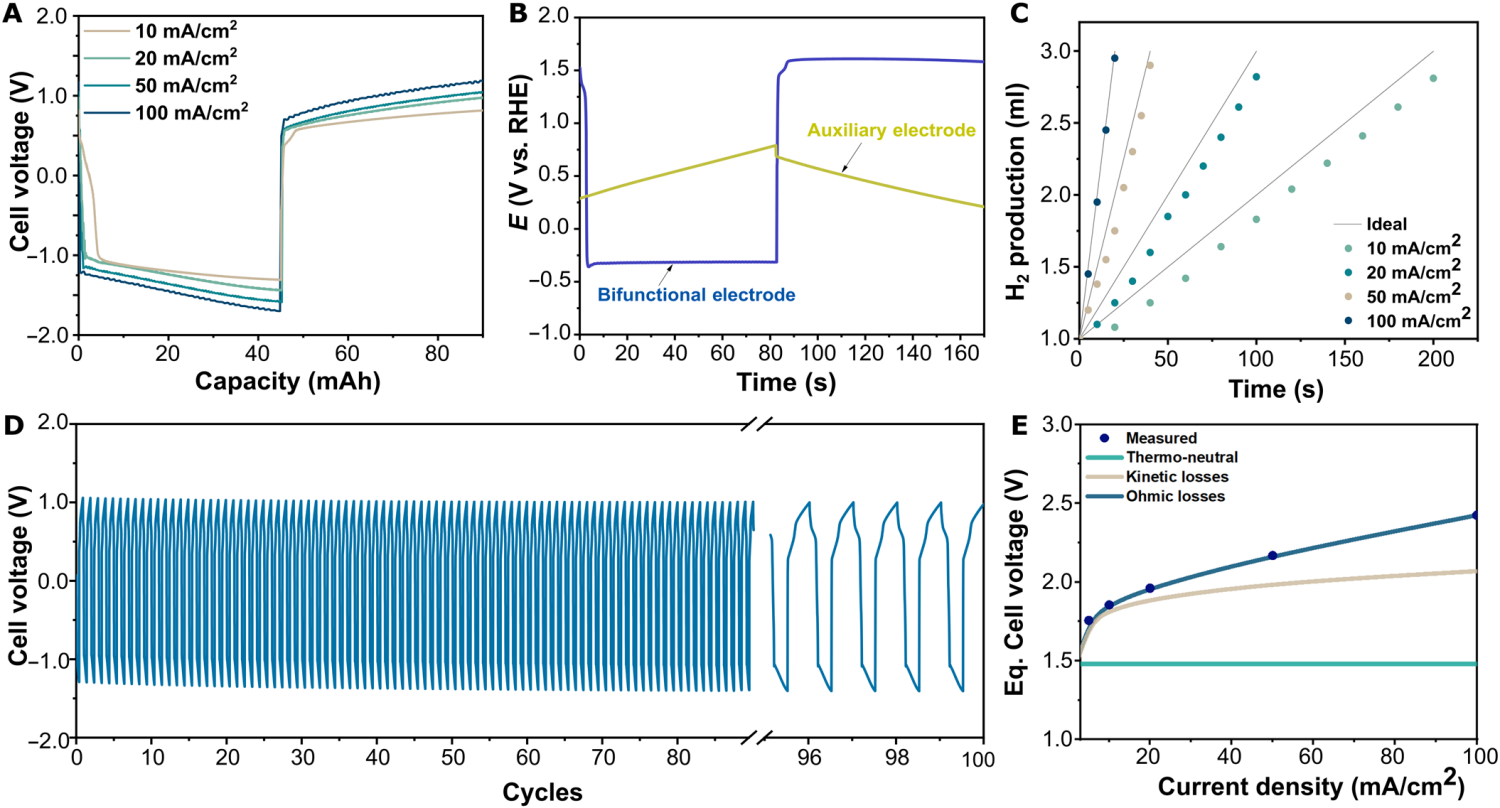
Performance and stability of SCE in a decoupled system. (**A**) Cell voltage profile of a water splitting test at different current densities in single-cell SCE in 1.0 M KOH. (**B**) Electrode potential of bifunctional and auxiliary electrodes during electrolysis. (**C**) Gas production during electrolysis at current densities ranging between 10 and 100 mA/cm^2^. (**D**) Stability test at 20 mA/cm^2^ in 5.0 M KOH. (**E**) Breakdown of different contributions to the equivalent cell voltage.

The water electrolysis process described in Fig. 1 (A and B) can be effectively carried out in a single cell. This approach results in an efficient and simplified setup to investigate the reaction mechanism and evaluate the performance of the electrochemical cell. An SCE was then constructed with CoFeP coated on Ni foam as a bifunctional electrocatalyst and ACC as an auxiliary electrode (with a loading mass of 10 mg/cm^2^). The cell voltage is recorded during constant current operation, indicating that the capacitive buildup during both the charging and discharging steps is shown in Fig. 4A. Notably, the initial voltage at each step is observed to be lower than the theoretical voltage for water electrolysis, indicative of the successful decoupling of the OER and HER. The observed cell voltage is attributed to the potential difference between the HER occurring on CoFeP surfaces and the polarized auxiliary electrode, and this potential difference is reversibly modulated during the charging and discharging steps. This was further studied by monitoring the electrode potentials in situ during water electrolysis at a current density of 50 mA/cm^2^, as shown in Fig. 4B. The evolution of electrode potential over time clearly indicates the galvanostatic charging of the auxiliary electrode, while the electrocatalyst shows a stable potential associated to hydrogen production during step 1 and subsequent oxygen evolution during step 2. An ohmic drop of 100 mV is observed when polarity is reversed in the transition between the charging and discharging steps. The overpotential obtained with CoFeP during electrochemical characterization corresponded well to the electrode potential observed during HER and OER, indicating a close correspondence between the SCE and the three-electrode setup. This facilitates the search for more bifunctional catalysts in the future. The cycle time during operation at 50 mA/cm^2^ is limited to 85 s to prevent the auxiliary electrode potential from reaching the highly oxidative region.

The produced hydrogen as determined by the Archimedes’ method (see Materials and Methods) during the charging step at different current densities is plotted in Fig. 4C. The evolution of hydrogen and suppression of oxygen evolution during charging step was confirmed by gas sensor detector using a selective H_2_ and O_2_ needle sensor (Unisense), as reported elsewhere (*48*). The gases were detected at the inlet of the H_2_ separation tank (see fig. S1B) in the liquid phase for improved detection of traces, and the recorded results of the continuous monitoring are shown in fig. S16. A steady increase in the dissolved hydrogen concentration is observed over consecutive cycles, whereas the oxygen concentration remains almost unchanged. Negligible increases in the oxygen concentration indicate that the chosen switching time of the valves allows effective separation of the gases without compromising the purity of the hydrogen produced. Upon scaling of the electrolyzer, a purging time could be necessary to ensure complete removal of the gases from the electrolyzer chamber and from the piping system. Thus, it is of great relevance to keep the volume upstream of the separation valves as small as possible to minimize the need for extended purging periods.

Moreover, the faradaic efficiency obtained at 10 mA/cm^2^ was 91%, which is associated to the time required for the electrocatalyst to transition between the two reactions, due to the polarization of the surface. When current was increased to 100 mA/cm^2^, faradaic efficiency reached up to 99%. The energy required during water electrolysis was calculated over the cyclic process (fig. S17). The energy consumed during the charging step is substantially lower than the theoretical value for every current density applied to the cell. However, the energy consumed during the discharging step does not come along with hydrogen production, leading to a steady increase of energy consumption. Overall, the energy requirement for hydrogen production at 10 mA/cm^2^ is 48 kWh/kg (69% energy efficiency based on lower heating value). The breakdown of the energy consumption was calculated from a simple 0D model (see Materials and Methods) and is presented in Fig. 4E. The contribution of the capacitive charging/discharging process can be assumed to be negligible compared to the equivalent cell voltage, because the process is completely reversible. From the curve fitting, it is clearly observed that the contribution of the ohmic losses is a substantial part in the system with a resistivity of 3.55 ohm/cm^2^, which is in good agreement with the Nyquist plot shown in fig. S18. The series resistance obtained is larger compared to that shown by Yan *et al.* (*34*) in a membrane-free architecture using NiOOH/Ni(OH)_2_ redox couple at similar electrolyte concentration and electrode distance. This suggests that the series resistance in our cell can be lowered by improving the electrode conductivity and by lowering the contact resistances. On the other hand, the contribution from kinetic losses arising from the overpotential needed for overall water splitting, is mainly associated to the sluggish kinetic of oxygen evolution during the discharging step.

Furthermore, the stability of the electrochemical cell was evaluated over 20 hours of operation (100 cycles) at a current density of 20 mA/cm^2^ in 5.0 M KOH, as shown in Fig. 4D. The voltage profile shows a reversibility in the two cycles, without appreciable increase in the final voltage neither during charging nor discharging that could indicate any loss of activity of the bifunctional catalyst or remanent potential build up on the auxiliary electrode. However, a transitionary state appears after switching polarity between the two steps that could arise due to a reversible redox reaction on the bifunctional catalyst, most likely hydroxide/oxyhydroxide transition that is probable in a highly alkaline environment and could be of interest to study in detail separately.

The performance of the SCE was compared to similar electrolyzer assemblies and the performance obtained through two-electrode characterization, as shown in table S1. The energy consumption obtained for the overall water splitting in a single-compartment experiment using the bifunctional catalyst (CoFeP) shows comparable cell voltage and efficiency values as noble metal–based catalyst and transition metal–based catalysts reported in literature (*49*, *50*). In the case of full cell assemblies (two-cell system in our case), the overall water splitting voltage at 10 mA/cm^2^ is substantially reduced compared to the system based on redox mediator (*24*, *51*). However, the efficiency values at this very early stage of development are slightly lower than proton exchange membrane water electrolyzer (PEMWE) or anion exchange membrane water electrolyzer (AEMWE) cells, with reported efficiencies of up to 65 to 70% at current densities ranging from 0.5 to 1 A/cm^2^ (*52*, *53*). Still, the extensive study of bifunctional electrocatalyst could provide a clear pathway to further develop this technology. In addition, to successfully transfer the electrocatalytic properties of the bifunctional materials to cell levels, the ohmic contribution and electrolyte flow distribution must be optimized to reduce the cell voltages at high current densities.

## DISCUSSION

In summary, we have proposed a decoupled water electrolyzer mediated by a capacitive storage mechanism, able to separately produce H_2_ and O_2_ in either time or space The concept was validated by assembling an SCE based on a transition metal–based phosphide bifunctional catalyst and a commercial carbon-based material achieving an equivalent cell voltage of 1.85 at a current density of 10 mA/cm^2^, with a good cycle stability without substantial increase in the cell voltage profile over extended periods of operation. The contribution of the different electrodes in the assembly was studied demonstrating the active role of auxiliary electrode. The storage capacity of the auxiliary electrode determines the cycle times to a large extent. Thus, increasing the materials capacitance or effective loading of capacitive material during the preparation of the auxiliary electrode could extend the cycle time, allowing the operation with higher current densities. On the other hand, the electrode conductivity plays a key role on the energy efficiency of the system due to the associated ohmic losses, which have a considerable contribution as observed from the breakdown of the equivalent cell voltage. The use of related capacitive or pseudocapacitive materials could potentially benefit the performance of the device. However, it is important to study the reversibility of the charging-discharging process, as well as carefully analyze the presence of irreversible redox waves, such as the one observed in acidic conditions for ACC, because this tends to reduce the reversibility of the process, limiting the overall device performance.

Improvements in the electrolyzer performance could also be achieved by a reduction of the overpotential for overall water splitting. Although some catalysts display excellent electrocatalytic activity toward the individual water splitting reactions, it must be considered that the same material must drive both the HER and OER reactions during the two stages of the electrolysis process, limiting the effectiveness of classical PGMs such as Ir- and Pt-based electrodes. In addition to the material selection, the electrolyzer design must ensure proper fluid distribution of the electrolyte to effectively allow electrocatalytic activity of the bifunctional electrode during the process on the electrodes. In addition, the bifunctional catalyst should not have any parasitic reaction upon reversing the current, because it would reduce the faradaic efficiency of the process by delaying the production of O_2_ and H_2_ at each stage, respectively. Thus, particular attention needs to be paid to the suppression of reversible side reactions on the catalyst surface that may hinder the production of hydrogen during the charging period.

The hybrid cell design introduces flexibility in the operation of an electrolyzer as water splitting in acidic or alkaline conditions can be carried out using similar system configuration. The electrolyzer can be operated in a single–half-cell configuration to provide intermittent production of hydrogen or in a two–half-cell configuration for continuous production of hydrogen, depending on the application. Thus, an electrolyzer stack can be constructed using similar design concept as conventional electrolyzers but replacing the membrane by a combination of a stainless-steel current collector and two auxiliary carbon electrodes stacked on each side to effectively separate the gas production with a proper manifold flow design. It is then of great relevance the use of cheap and readily available components as auxiliary electrode and current collector; such is the case of the system proposed in this work.

This work is a first attempt to shift the paradigm of conventional electrolysis technologies toward capacitive mediated electrocatalytic reactions, introducing flexibility and allowing the use of earth-abundant materials such as carbon to bring down the costs of water splitting technology that is adapted to the fluctuating loads in renewable electrical power with very little chances of mixing of hydrogen and oxygen gases, thus avoiding potential explosions.

## MATERIALS AND METHODS

### Materials and reagents

Hydrochloric acid 35% (HCl), ethanol, and nitric acid (HNO_3_) were used for cleaning the substrate of current collectors and the electrodes. Hexachloroplatinic acid (H_2_PtCl_6_), ethylene glycol, sodium hypophosphite (NaH_2_PO_2_), nickel(II) chloride (NiCl_2_), cobalt(II) chloride (CoCl_2_), and ammonium chloride (NH_4_Cl) were used for the synthesis of electrocatalytic material. Ninety-eight percent sulfuric acid (H_2_SO_4_) and potassium hydroxide (KOH) pellets were used for the electrolytes during the electrolyzer evaluation. All the reagents and chemicals used in this work were purchased from Sigma-Aldrich Chemie GmbH (Taufkirchen, Munich, Germany) and used without further purification. Zorflex FM10 ACC was supplied by Chemviron Carbon Ltd. (UK), and graphite sheet with a thickness of 0.18 mm was obtained from Mineral Seal Corporation (Minseal, USA). Nickel Foam was supplied by Xinxiang purification equipment Ltd. (China).

### Material synthesis

#### Preparation of platinum nanoparticles supported on carbon

Platinum nanoparticles supported on graphite sheet (Pt/C) were synthesized by a polyol method as reported by Fiévet *et al.* (*54*). Briefly, 1 ml of ethanol solution containing H_2_PtCl_4_ of 1000 parts per million was prepared and added dropwise to graphite substrate (5 cm by 5 cm) and dried at 60°C. The graphite substrate was immersed in 150 ml of ethylene glycol at 150°C for 10 min and rinsed thoroughly with deionized water and dried in atmospheric oven at 60°C before the deposition of platinum nanoparticles.

#### Preparation of cobalt-iron phosphide supported on nickel foam

CoFeP was grown on Ni foam substrate to provide a 3D network and to provide good electrical conductivity. The synthesis was conducted by galvanostatic electrodeposition. Before electrode preparation, the Ni foam was sonicated in 0.1 M HCl for 15 min followed by ethanol and deionized water sonication and rinsing. The as-cleaned substrates were immersed in a growth solution containing 100 mM sodium hypophosphite (NaH_2_PO_2_) as precursor for phosphorous (P), 200 mM NH_4_Cl, and a 20 mM equimolar concentration of iron(II) sulfate (FeSO_4_) and cobalt(II) chloride (CoCl_2_) as the metal precursor. Electrodeposition was carried out by applying constant current of 5 mA/cm^2^ for 60 s followed by 600 s with 50 mA/cm^2^. After deposition, the electrode was removed and thoroughly washed with deionized water and dried in vacuum oven at 60°C. Control samples of CoP and FeP were prepared following the same procedure, maintaining the metal concentration at 10 mM.

### Material characterization

Field emission scanning electron microscope (Ultra 55, ZEISS) was used to examine the morphology of the synthesized electrocatalyst and the surface roughness of electrodes. XPS experiments were performed in a VG-Microtech Multilab 3000 electron spectrometer (VG Scientific, Sussex, UK) equipped with a hemispherical electron analyzer with nine channeltrons (passing energy of 2 to 200 eV) and an x-ray source with Al radiation (Kα at 1253.6 eV). The bond energy of the C1s peak at 284.6 eV was taken as an internal standard. The XPS data were deconvoluted using the XPSPEAK41 program, and the experimental curves were fitted using Lorentz-Gaussian functions, and the background was a Shirley line. The XPS data were collected over seven cycles to calculate an average, with an estimated uncertainty of ±5%. Porous structure and surface area were studied by N_2_ adsorption at −196°C using an Autosorb-6B (Quantachrome) equipment after degassing the samples at 200°C for 6 hours. BET method was used to calculate the surface area and the pore size distribution using SAIEUS software based on 2D non-local density functional theory (2D-NLDFT) heterogeneous surface model. Metal content in the structure of the bifunctional catalyst was determined using ICP-OES (Thermo Scientific iCAP 6500).

### Electrochemical characterization

Electrochemical characterization of active and auxiliary electrodes was conducted in a three-electrode conventional electrochemical workstation using a potenciostat/galvanostat Gamry Interface 1010E. The three-electrode setup consisted of Hg/HgO and Hg/HgCl_2_ as reference electrodes for measurements in alkaline and acidic electrolytes, respectively, while carbon was the counter electrode, and the electrocatalyst or the capacitive electrode were used as working electrode. The electrochemical characterization was performed on three different samples for each electrocatalyst to evaluate the reproducibility of the synthesis method. In addition, each sample was tested three times after a stable response was achieved to determine the uncertainty of the measurements. All the potentials are reported in the RHE and were converted from Hg/HgCl_2_ and Hg/HgO using Eqs. 1 and 2, respectively (*55*, *56*)

$$E_{\mathrm{RHE}}=E_{\mathrm{Hg}/{\mathrm{HgCl}}_{2}}+0.059\;\mathrm{pH}+0.241$$ (1)

$$E_{\mathrm{RHE}}=E_{\mathrm{Hg}/\mathrm{HgO}}+0.059\;\mathrm{pH}+0.098$$ (2)

CV was conducted to determine the charge storage in auxiliary electrode in the potential region of water stability (0–1.2 V) at different scan rates varying from 5 to 100 mV/s, while LSV was conducted at a scan rate of 5 mV/s. Voltammetric measurements were iR-corrected using the current interrupt method. GCD was used in the range of 0.25 to 10 A/g to study the electrochemical process taking place on each electrode during electrolysis. Long-term stability of the catalyst under operational conditions was also evaluated by GCD over 100 cycles. Last, electrochemical impedance spectroscopy was used not only to further study the electrochemical response of electrodes but also to determine the resistive component of the electrolyzer assembly. Impedance measurements were recorded in the frequency range from 100 kHz to 10 mHz at open circuit potential. ECSA was estimated from the capacitance of the double layer *C*_dl_ obtained from the current density of CV measurements at different current densities in a non-faradaic potential window. The *C*_dl_ was calculated following Eq. 3

$$C_{\mathrm{dl}}=\frac{∆I_{\mathrm{dl}}}{w}$$ (3)

where Δ*I*_dl_ is the current density difference as obtained in fig. S11 and *w* is the scan rate used for CV measurements.

### Water electrolysis experiments

Water electrolysis system is composed of two identical cells with a compartment of 10 cm by 10 cm and flow through architecture, as shown in fig. S1. The cell assembly consists of a bifunctional electrode, a capacitive electrode, a cellulose-based filter paper Whatman no. 1 (thickness of 200 μm) used as spacer placed in between the electrodes to electrically isolate both electrodes, stainless steel (AISI 316 L) current collectors for each electrode, and the enclosing components (end plates and gasket). The input energy was provided by a benchtop power supply, and the applied current was controlled with a custom-built Arduino circuit to automatically switch between the charging and discharging mode as a function of predefined times. Current and voltage were recorded in a digital multimeter Keithley 2110. Conductivity of the electrolyte was monitored continuously using a Dip-In probe ET915 placed at the outlet and connected to an EPU 357 IsoPod (eDAQ). Gas production was quantified by determining the volume displaced in a closed water reservoir at atmospheric pressure as shown in fig. S1. Gas evolved at different steps of the operation cycle were separated by a three-way valve connected to the recirculation system to accumulate hydrogen and oxygen in separate containers for further study, as schematically described in fig. S1 (B and C). Hydrogen and oxygen were first detected for qualitative analysis in aqueous and gas phases using Unisense microsensor setup is composed of a H_2_ and O_2_ needle sensor connected to a H_2_ UniAmp unit and O_2_ UniAmp unit, respectively, similar to detection setup reported elsewhere (*48*).

The energy consumption (*E*_cons_) and equivalent cell voltage (*U*_Eq. Cell_) were calculated following Eqs. 4 and 5, respectively

$$E_{\text{cons}}=\frac{\int_{0}^{t_{\text{cycle}}}I\;U_{\text{Cell}}\;\mathrm{dt}}{V_{H_{2}}}$$ (4)

$$U_{\mathrm{Eq}.\text{Cell}}=\frac{\int_{0}^{t_{\text{cycle}}}U\;\mathrm{dt}}{t_{\text{charg}}}$$ (5)

where *I* is the current applied to the SCE, *U*_cell_ is the cell voltage recorded over time, $V_{H_{2}}$ is the volume of hydrogen per cycle, *t*_cycle_ is the cycle time considering both steps, and *t*_charg_ is the time of the charging step.

The different contributions to the equivalent cell voltage *V*(*i*) were identified by using a simplified 0D model. The voltage can be described as in Eq. 6

$$V\;\left(i\right)=U+∆V_{\text{kinetic}}\left(i\right)+∆V_{\text{ohmic}}\left(i\right)$$ (6)

where *U* is the equilibrium cell voltage, Δ*V*_kinetic_ corresponds to the kinetic losses, and Δ*V*_ohmic_ corresponds to the ohmic losses due to the resistive component within the cell. The contribution of ohmic and kinetic losses is described in Eqs. 7 and 8, respectively

$$∆V_{\text{ohmic}}\left(i\right)=i∙R$$ (7)

$$∆V_{\text{kinetic}}\left(i\right)=b∙\text{log}\left(\frac{i}{i_{0}}\right)$$ (8)

where *R* corresponds to the cell series resistance, *i*_0_ is the exchange current density, and *b* is the Tafel slope. Thus, following Eq. 6, we can represent the equivalent cell voltage through Eq. 9

$$V\left(i\right)=U+i∙R+b∙\text{log}\left(\frac{i}{i_{0}}\right)$$ (9)

## Supplementary Materials

### This PDF file includes:

Figs. S1 to S18 References
