# Supplementary material for: Decoupled supercapacitive electrolyzer for membrane-free water splitting
Source: Sci Adv. 2024 Mar 6;10(10):eadi3180. doi: 10.1126/sciadv.adi3180 (PMC10917338; doi:10.1126/sciadv.adi3180)
Supplement: Supplementary file 1 — Figs. S1 to S18 References [file sciadv.adi3180_sm.pdf]

Science Advances
AAAS

Supplementary Materials for

Decoupled supercapacitive electrolyzer for membrane-free water splitting

Esteban A. Toledo-Carrillo *et al.*

Corresponding author: Esteban A. Toledo-Carrillo, eatc@kth.se; Joydeep Dutta, joydeep@kth.se

Sci. Adv. **10**, eadi3180 (2024)
DOI: 10.1126/sciadv.adi3180

This PDF file includes:

Figs. S1 to S18
References

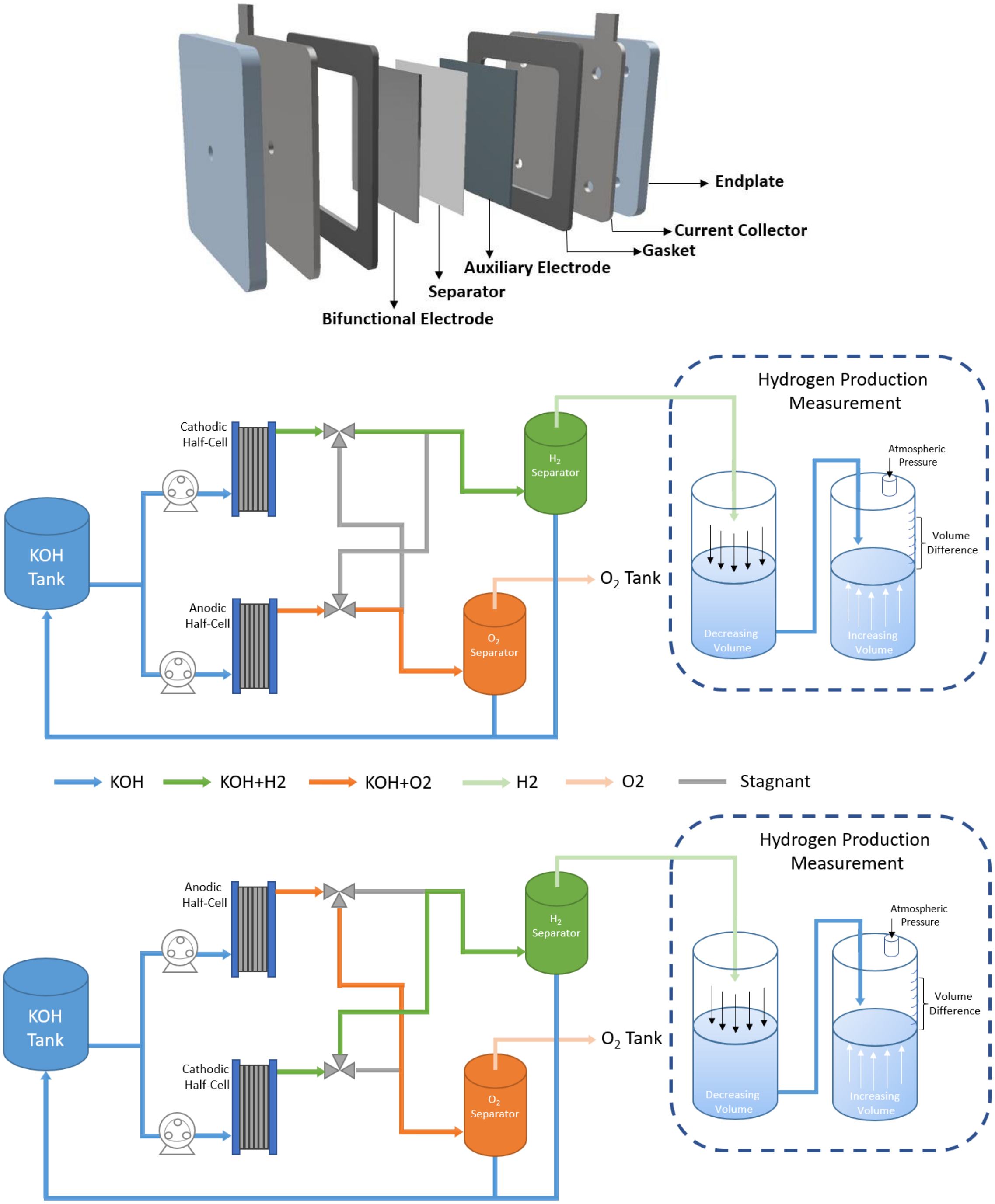

Fig. S1. Schematic representation of cell assembly and balance of plant. (**A**) Detailed construction of the cells in the supercapacitive electrolyzer. Schematic representation of the setup used for electrolysis experiments including hydrogen production quantification during (**B**) charging and (**C**) discharging steps.

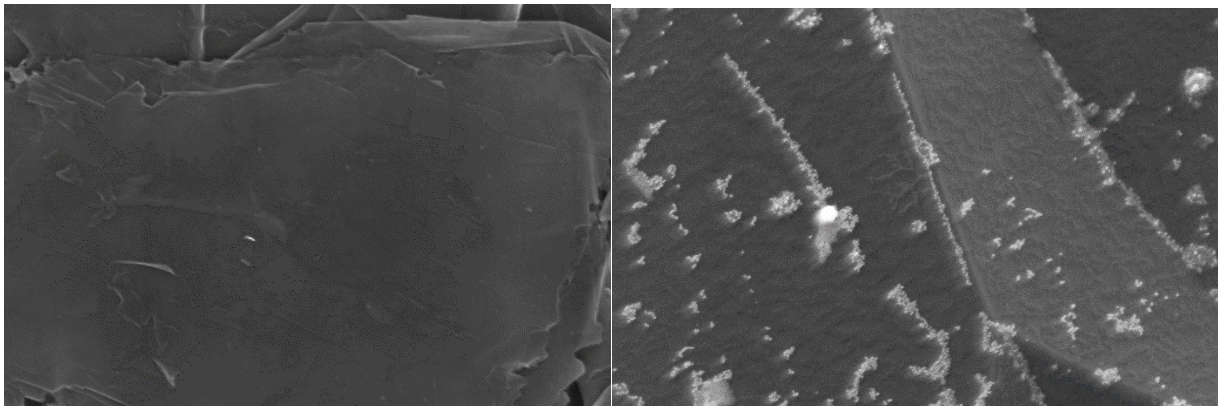

Fig. S2. Morphology of Pt/C electrodes. Scanning electron microscopy image of pristine graphite substrate and Pt nanoparticle deposited on graphite.

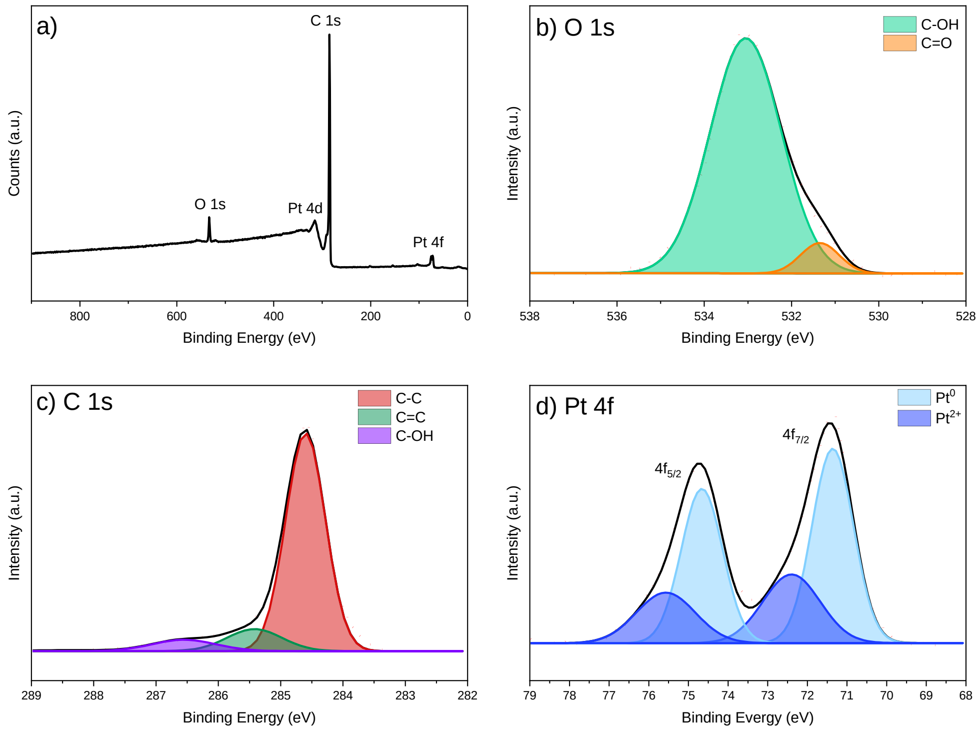

Fig. S3. X-ray photoelectron spectroscopy study of Pt/C electrode. (**A**) XPS survey spectra. High-resolution XPS spectra of (**B**) O 1s, (**C**) C 1s and (**D**) Pt 4f.

Figure S3c displays X-ray photoelectron spectroscopy (XPS) deconvoluted spectra of carbon. The C 1s spectrum was resolved into three distinct signals at binding energies of 284.6 eV, 285.4 eV, and 286.6 eV, corresponding to C-C, C=C, and C-OH functional groups, respectively. The O 1s spectrum (**Figure S3b**) exhibited two signals at 531.4 eV and 533.0 eV, attributed to C=O and C-OH groups, respectively (57, *58*). The Pt 4f spectrum (**Figure S3d**) revealed two peaks, representing the spin-orbital splitting of $4f_{7/2}$ and $4f_{5/2}$ photoemission lines. The $4f_{7/2}$ signal was deconvoluted into two peaks centered at 71.4 eV and 72.4 eV, corresponding to Pt^0 and Pt^{2+} oxidation states, respectively. Similarly, the $4f_{5/2}$ peak was also resolved into two signals centered at 74.7 eV and 75.6 eV for Pt^0 and Pt^{2+} oxidation states, respectively. The relative atomic concentration of Pt^0 and Pt^{2+} was estimated using the peak areas, which were found to be 67.7 % and 32.3 %, respectively.

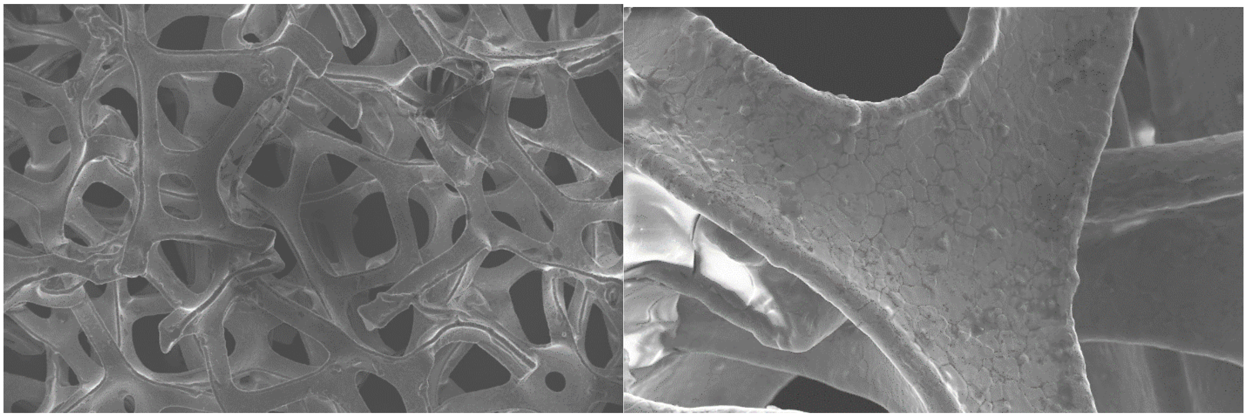

Fig. S4. Morphology of pristine nickel foam. Scanning electron microscopy image of pristine nickel (Ni) Foam.

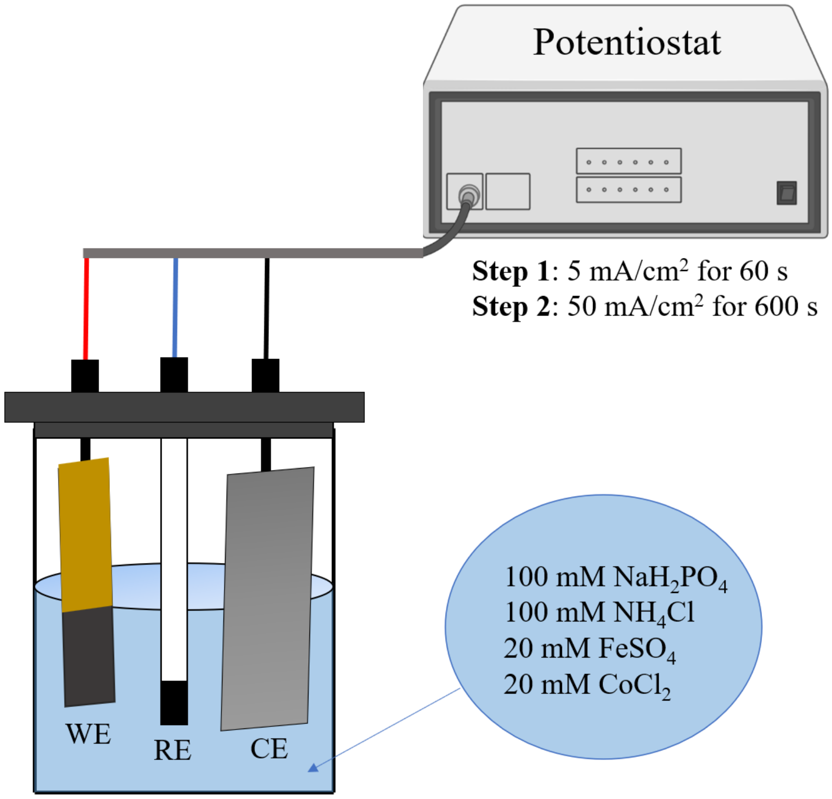

Fig. S5. Electrode preparation configuration. Schematic representation of setup used for electrodeposition.

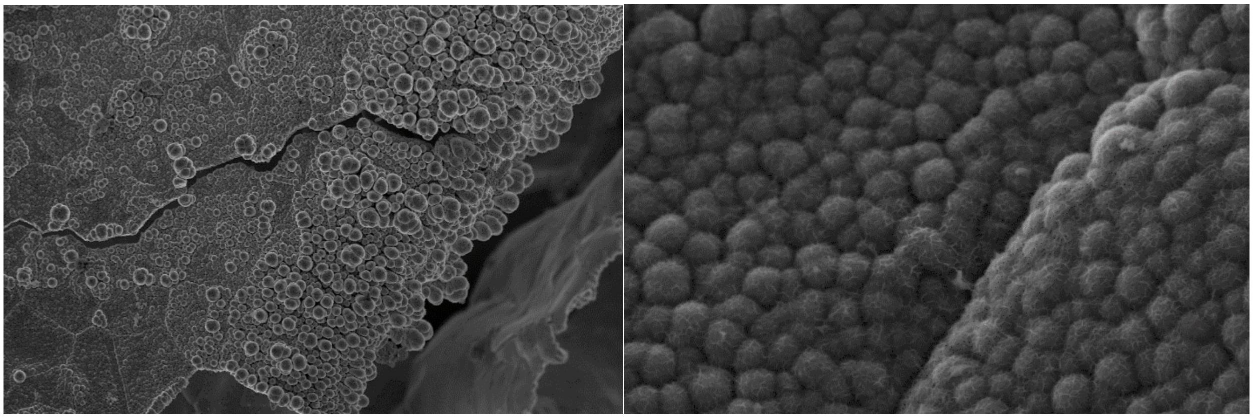

Fig. S6. Morphology of cobalt phosphide electrode. Scanning electron micrograph of cobalt phosphide (CoP) coatings on Ni Foam substrates.

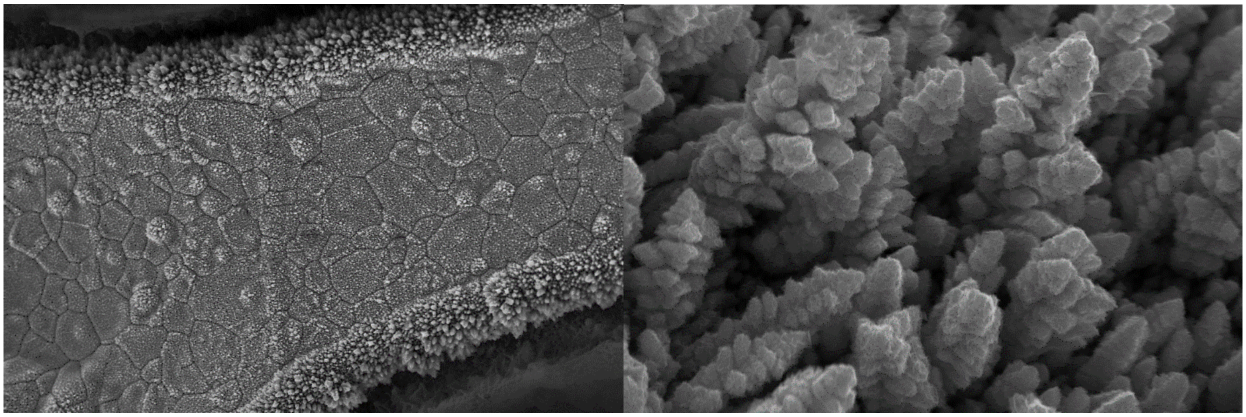

Fig. S7. Morphology of iron phosphide electrode. Scanning electron micrograph of iron phosphide (FeP) coatings on Ni Foam substrates.

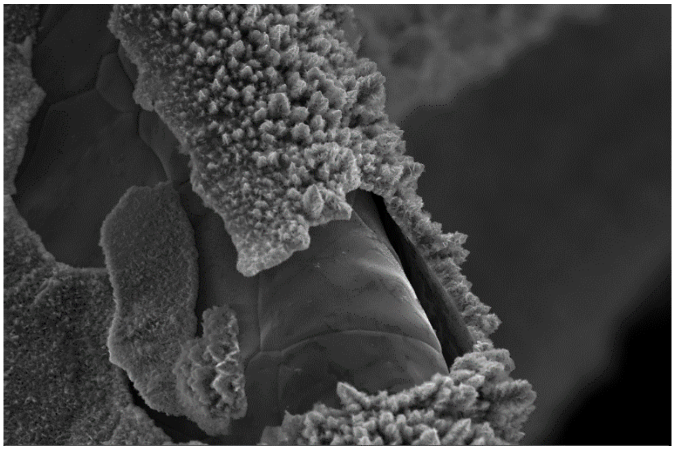

Fig. S8. Determination of cobalt iron phosphide film thickness. Scanning electron micrograph of cobalt iron phosphide (CoFeP) coatings on Ni Foam substrates after sonication.

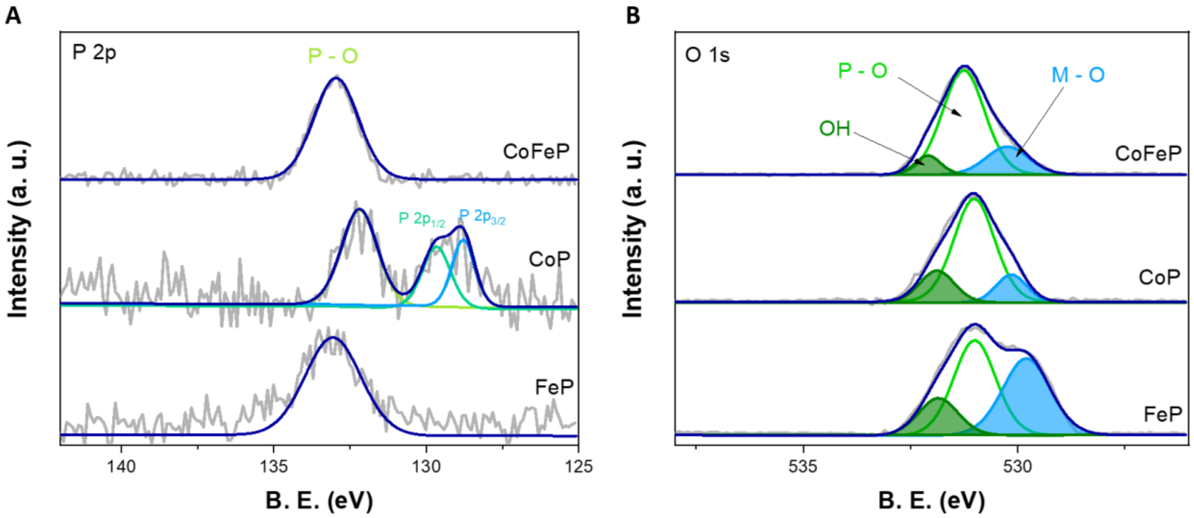

Fig. S9. X-ray photoelectron spectroscopy study of electrodeposited electrode. (**A**) P 2p XPS spectra and (**B**) O 1s spectra for cobalt iron phosphide (CoFeP), cobalt phosphide (CoP) and iron phosphide (FeP) samples.

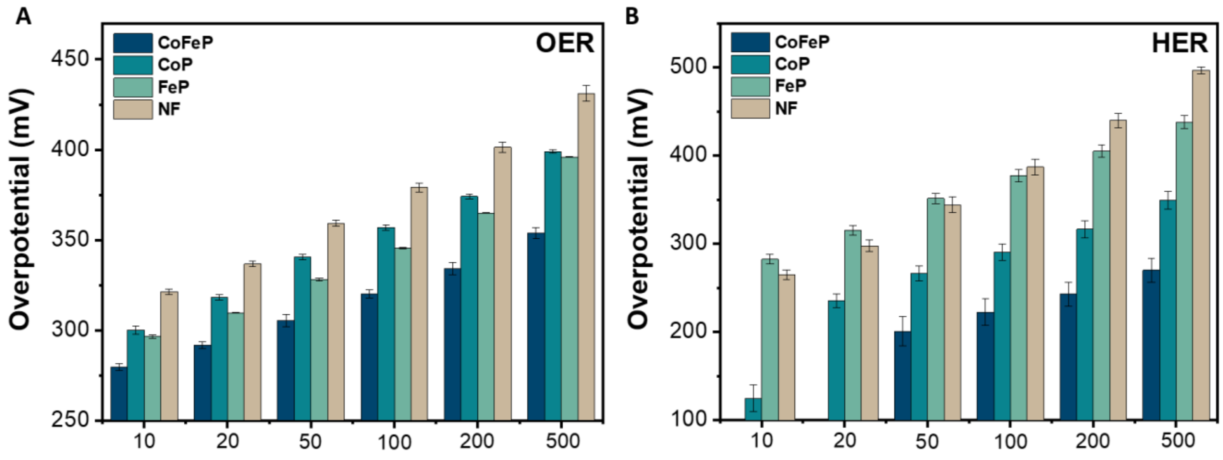

Fig. S10. Water splitting overpotentials of electrodeposited electrodes. (**A**) Oxygen Evolution Reaction (OER) overpotential and (**B**) Hydrogen Evolution Reaction (HER) overpotential at 10, 20, 50, 100, 200 and 500 mA/cm^2 of the synthesized cobalt iron phosphide (CoFeP), cobalt phosphide (CoP) and iron phosphide (FeP) catalysts and Ni foam substrates.

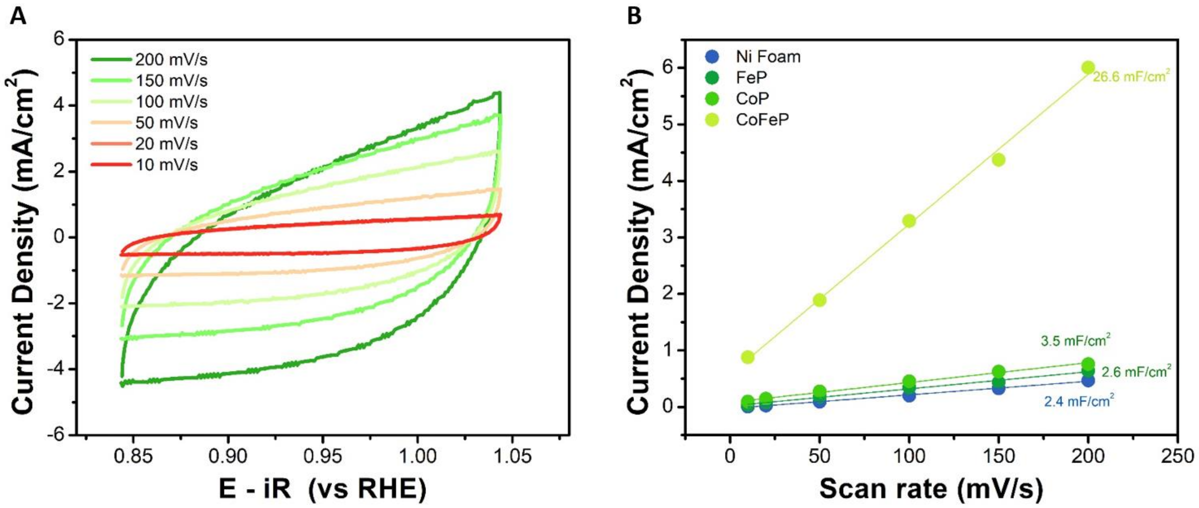

Fig. S11. Comparison of double layer capacitance of electrodeposited phosphide electrodes. (**A**) Cyclic voltammetry of cobalt iron phosphide (CoFeP) coated Ni Foam in a non-faradaic region at different scan rate in 1.0 M KOH (**B**) C_{dl} calculated from the I_{dl} obtained from CV measurements.

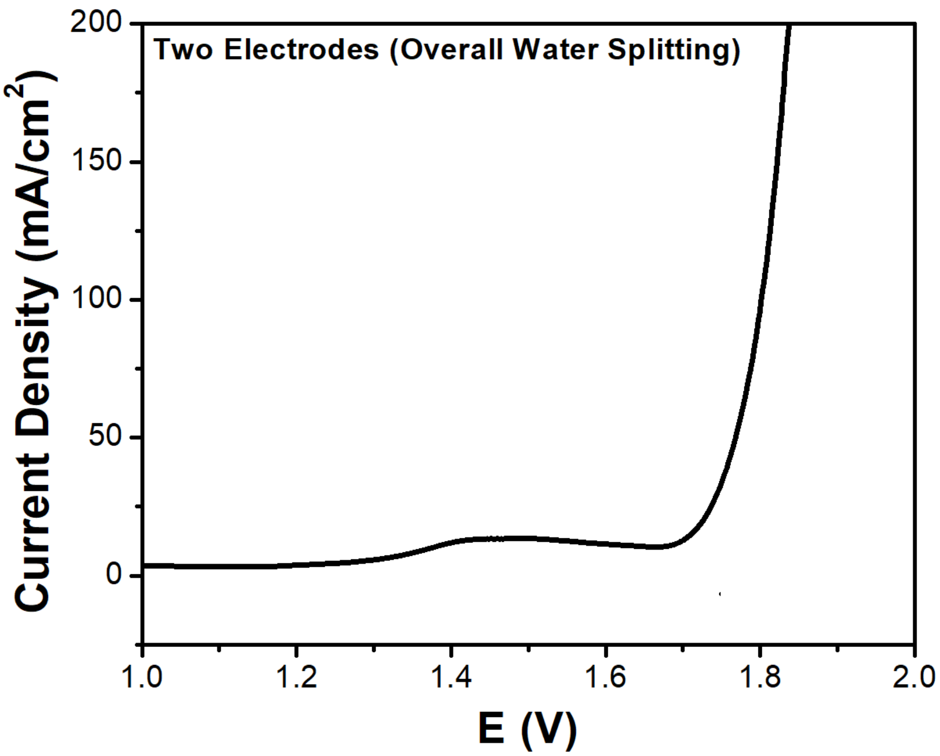

Fig. S12. Overall water splitting activity of cobalt iron phosphide electrode. Linear sweep voltammetry (LSV) of overall water splitting performance at 5 mV/s in a two-electrode setup with cobalt iron phosphide (CoFeP) coated Ni Foam substrate.

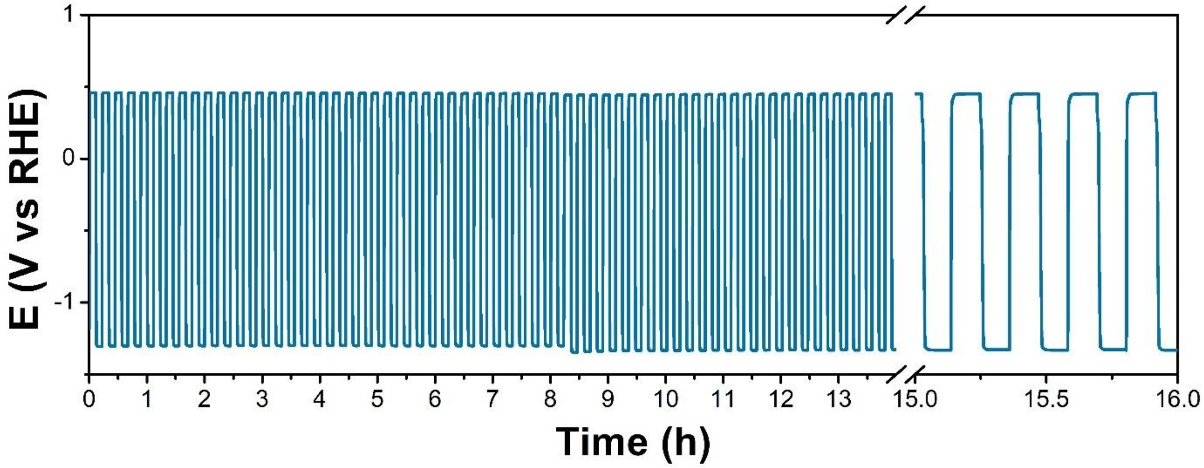

Fig. S13. Stability of cobalt iron phosphide. Long-term test of cobalt iron phosphide (CoFeP) coated Ni Foam substrate over 16 h (75 cycles) of galvanostatic charge and discharge at 20 mA/cm^2.

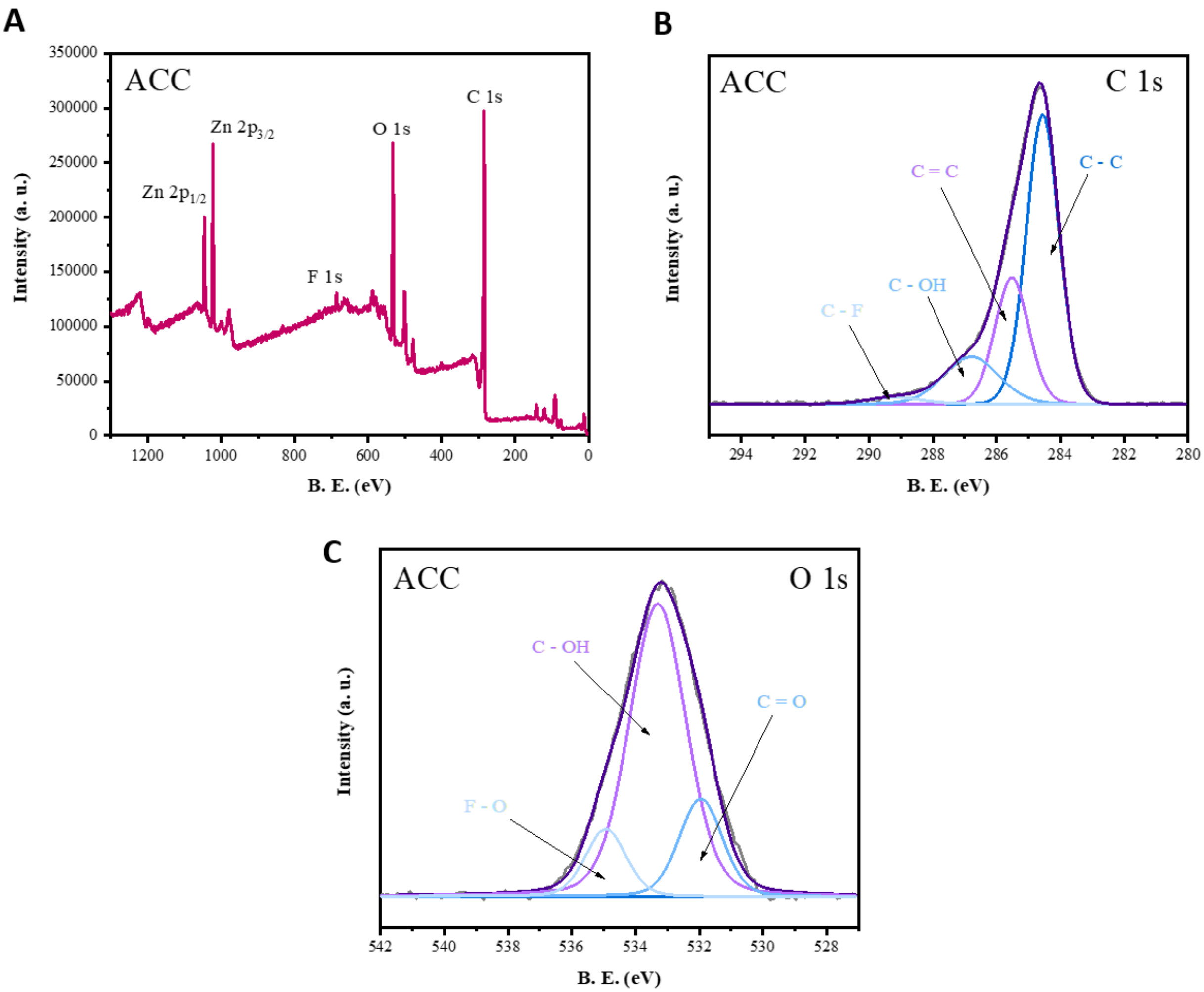

Fig. S14. X-ray photoelectron spectroscopy study of activated carbon cloth. (**A**) XPS survey spectra, (**B**) high-resolution C 1s spectra and **(C)** high-resolution O 1s spectra of activated carbon cloth (ACC) capacitive electrode.

The XPS analysis of the ACC sample is presented below. The survey scan of the sample (**Figure S14a)** is noteworthy, as commercial carbon cloth often contain significant impurities. The primary orbitals detected in the survey scan are C 1s, O 1s, F 1s, and Zn 2p, with several additional peaks that are difficult to assign without further information from the manufacturer. The predominant impurity present in the sample is Zn, but it is found at low atomic percentages of 4.0%.

Figure S14b presents the deconvolution of the C 1s spectrum of ACC, displaying four distinct peaks corresponding to C-C (284.6 eV), C=C (285.5 eV), C-OH (286.8 eV), and C=O (289.0 eV) functional groups. The impurities present in the sample do not exhibit appreciable contributions, as their relative ratio is significantly lower than that of the bonds observed in **Figure S14b**. The

deconvolution of the O 1s spectrum (**Figure S14c**) reveals three distinct contributions at 532.0 eV, 533.3 eV, and 534.9 eV, corresponding to the C=O, C-OH, respectively. The major component is associated with the C-OH bond, followed by the C=O bond.

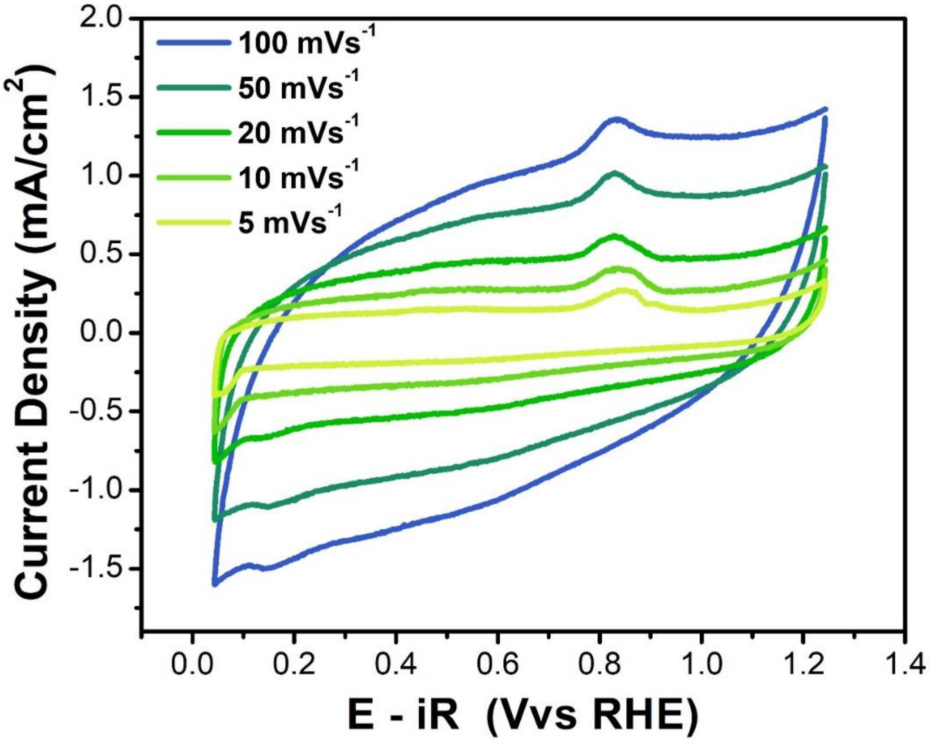

Fig. S15. Cyclic voltammogram of activated carbon cloth. Cyclic voltammogram of activated carbon cloth (ACC) at different scan rates in the potential windows for water splitting.

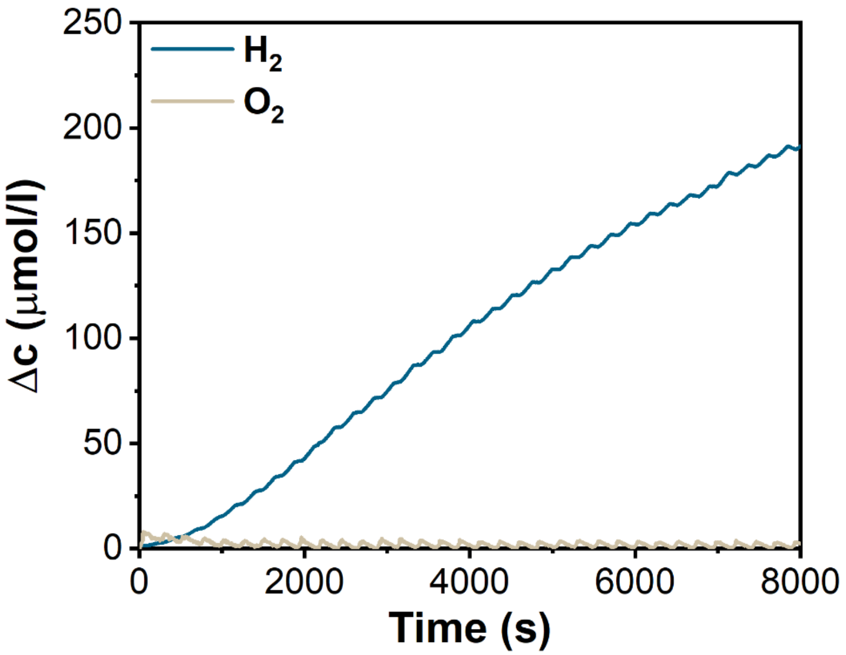

Fig. S16. Gas separation during decoupled electrolysis. Dissolved hydrogen and oxygen concentration changes in the liquid phase over consecutive charging and discharging cycles.

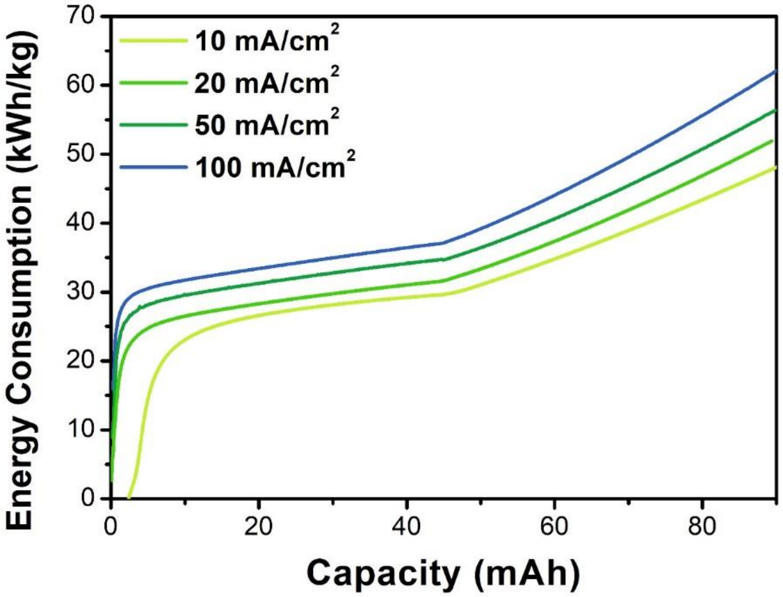

Fig. S17. Energy consumption profile of a supercapacitive electrolyzer. Energy calculated from cell voltage profile of an electrolyzer assembly with cobalt iron phosphide (CoFeP) and activated carbon cloth (ACC) operated at current densities from 10 – 100 mA/cm^2.

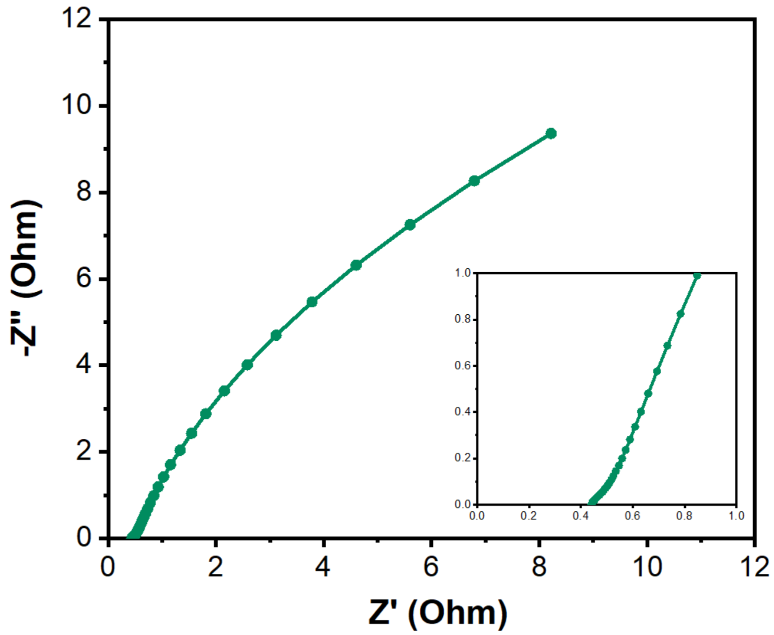

Fig. S18. Impedance measurement of a half-cell SCE assembly. Nyquist plot of impedance measurement of the SCE, showing the high frequency resistance (HFR) of the device in the intercept with the real axis.

REFERENCES

1. D. Bogdanov, J. Farfan, K. Sadovskaia, A. Aghahosseini, M. Child, A. Gulagi, A. S. Oyewo, L. de Souza Noel Simas Barbosa, C. Breyer, Radical transformation pathway towards sustainable electricity via evolutionary *steps Nat. Commun.* **10**, 1077 (2019).

2. M. van der Spek, C. Banet, C. Bauer, P. Gabrielli, W. Goldthorpe, M. Mazzotti, S. T. Munkejord, N. A. Røkke, N. Shah, N. Sunny, D. Sutter, J. M. Trusler, M. Gazzani, Perspective on the hydrogen economy as a pathway to reach net-zero CO_2 emissions in Europe. *Energ. Environ. Sci.* **15**, 1034–1077 (2022).

3. X. Yang, C. P. Nielsen, S. Song, M. B. McElroy, Breaking the hard-to-abate bottleneck in China's path to carbon neutrality with clean hydrogen. *Nat. Energy* **7**, 955–965 (2022).

4. W. Liu, H. Zuo, J. Wang, Q. Xue, B. Ren, F. Yang, The production and application of hydrogen in steel industry. *Int. J. Hydrogen Energy* **46**, 10548–10569 (2021).

5. F. Dawood, M. Anda, G. M. Shafiullah, Hydrogen production for energy: An overview. *Int. J. Hydrogen Energy* **45**, 3847–3869 (2020).

6. T. da Silva Veras, T. S. Mozer, D. da Costa Rubim Messeder dos Santos, A. da Silva César, Hydrogen: Trends, production and characterization of the main process worldwide. *Int. J. Hydrogen Energy* **42**, 2018–2033 (2017).

7. H. Zhang, Z. Sun, Y. H. Hu, Steam reforming of methane: Current states of catalyst design and process upgrading. *Renew. Sustain. Energy Rev.* **149**, 111330 (2021).

8. D. Oudejans, M. Offidani, A. Constantinou, S. Albonetti, N. Dimitratos, A. Bansode, A comprehensive review on two-step thermochemical water splitting for hydrogen production in a redox cycle. *Energies* **15**, 3044 (2022).

9. S. Cao, L. Piao, X. Chen, Emerging photocatalysts for hydrogen evolution. *Trends Chem.* **2**, 57–70 (2020).

10. P. Ganguly, M. Harb, Z. Cao, L. Cavallo, A. Breen, S. Dervin, D. D. Dionysiou, S. C. Pillai, 2D nanomaterials for photocatalytic hydrogen production. *ACS Energy Lett.* **4**, 1687–1709 (2019).

11. M. Ahmed, I. Dincer, A review on photoelectrochemical hydrogen production systems: Challenges and future directions. *Int. J. Hydrogen Energy* **44**, 2474–2507 (2019).

12. S. D. Tilley, Recent advances and emerging trends in photo-electrochemical solar energy conversion. *Adv. Energy Mater.* **9**, 1802877 (2018).

13. M. Chatenet, B. G. Pollet, D. R. Dekel, F. Dionigi, J. Deseure, P. Millet, R. D. Braatz, M. Z. Bazant, M. Eikerling, I. Staffell, P. Balcombe, Y. Shao-Horn, H. Schäfer, Water electrolysis: From textbook knowledge to the latest scientific strategies and industrial developments. *Chem. Soc. Rev.* **51**, 4583–4762 (2022).

14. Y. Xu, C. Wang, Y. Huang, J. Fu, Recent advances in electrocatalysts for neutral and large-current-density water electrolysis. *Nano Energy* **80**, 105545 (2021).

15. S. Trasatti, Water electrolysis: Who first? *J. Electroanal. Chem.* **476**, 90–91 (1999).

16. B. Bensmann, R. Hanke-Rauschenbach, K. Sundmacher, In-situ measurement of hydrogen crossover in polymer electrolyte membrane water electrolysis. *Int. J. Hydrogen Energy* **39**, 49–53 (2014).

17. A. G. Wallace, M. D. Symes, Decoupling strategies in electrochemical water splitting and beyond. *Joule* **2**, 1390–1395 (2018).

18. X. Liu, J. Chi, B. Dong, Y. Sun, Recent progress in decoupled H_2 and O_2 production from electrolytic water splitting. *ChemElectroChem* **6**, 2157–2166 (2019).

19. J. Huang, Y. Wang, Efficient renewable-to-hydrogen conversion via decoupled electrochemical water splitting. *Cell Rep.* **1**, 100138 (2020).

20. P. J. McHugh, A. D. Stergiou, M. D. Symes, Decoupled electrochemical water splitting: From fundamentals to applications. *Adv. Energy Mater.* **10**, 2002453 (2020).

21. Z. P. Ifkovits, J. M. Evans, M. C. Meier, K. M. Papadantonakis, N. S. Lewis, Decoupled electrochemical water-splitting systems: A review and perspective. *Energ. Environ. Sci.* **14**, 4740–4759 (2021).

22. A. Paul, M. D. Symes, Decoupled electrolysis for water splitting. *Curr. Opin. Green Sustain. Chem.* **29**, 100453 (2021).

23. A. Landman, A. Rothschild, G. S. Grader, "New electrolyzer principles: Decoupled water splitting" in *Electrochemical Power Sources: Fundamentals, Systems, and Applications*, T. Smolinka, J. Garche, Eds. (Elsevier, 2022), chap. 12, pp. 407–454.

24. M. D. Symes, L. Cronin, Decoupling hydrogen and oxygen evolution during electrolytic water splitting using an electron-coupled-proton buffer. *Nat. Chem.* **5**, 403–409 (2013).

25. J. Lei, J.-J. Yang, T. Liu, R.-M. Yuan, D.-R. Deng, M.-S. Zheng, J.-J. Chen, L. Cronin, Q.-F. Dong, Tuning redox active polyoxometalates for efficient electron-coupled proton-buffer-mediated water splitting *Chem.–Eur. J.* **25**, 11432–11436 (2019).

26. F. Wang, H. Sheng, W. Li, J. B. Gerken, S. Jin, S. S. Stahl, Stable Tetrasubstituted Quinone Redox Reservoir for Enhancing Decoupled Hydrogen and Oxygen Evolution. *ACS Energy Lett.* **6**, 1533–1539 (2021).

27. F. Zhang, H. Zhang, M. Salla, N. Qin, M. Gao, Y. Ji, S. Huang, S. Wu, R. Zhang, Z. Lu, Q. Wang, Decoupled redox catalytic hydrogen production with a robust electrolyte-borne electron and proton carrier. *J. Am. Chem. Soc.* **143**, 223–231 (2021).

28. W. Li, N. Jiang, B. Hu, X. Liu, F. Song, G. Han, T. J. Jordan, T. B. Hanson, T. L. Liu, Y. Sun, Electrolyzer design for flexible decoupled water splitting and organic upgrading with electron reservoirs. *Chem* **4**, 637–649 (2018).

29. J. Lee, M. Kim, Y.-T. Kim, J. Choi, Asymmetric cell design for decoupled hydrogen and oxygen evolution paired with V(II)/V(III) redox mediator. *Catal. Today* **403**, 67–73 (2022).

30. B. Rausch, M. D. Symes, L. Cronin, A Bio-Inspired, A bio-inspired, small molecule electron-coupled-proton buffer for decoupling the half-reactions of electrolytic water splitting. *J. Am. Chem. Soc.* **135**, 13656–13659 (2013).

31. B. Rausch, M. D. Symes, G. Chisholm, L. Cronin, Decoupled catalytic hydrogen evolution from a molecular metal oxide redox mediator in water splitting. *Science* **345**, 1326–1330 (2014).

32. L. Chen, X. Dong, Y. Wang, Y. Xia, Separating hydrogen and oxygen evolution in alkaline water electrolysis using nickel hydroxide. *Nat. Commun.* **7**, 11741 (2016).

33. H. Dotan, A. Landman, S. W. Sheehan, K. D. Malviya, G. E. Shter, D. A. Grave, Z. Arzi, N. Yehudai, M. Halabi, N. Gal, N. Hadari, C. Cohen, A. Rothschild, G. S. Grader, Decoupled hydrogen and oxygen evolution by a two-step electrochemical–chemical cycle for efficient overall water splitting. *Nat. Energy* **4**, 786–795 (2019).

34. X. Yan, J. Biemolt, K. Zhao, Y. Zhao, X. Cao, Y. Yang, X. Wu, G. Rothenberg, N. Yan, A membrane-free flow electrolyzer operating at high current density using earth-abundant catalysts for water splitting. *Nat. Commun.* **12**, 4143 (2021).

35. M. Vanags, G. Kulikovskis, J. Kostjukovs, L. Jekabsons, A. Sarakovskis, K. Smits, L. Bikse, A. Šutka, Membrane-less amphoteric decoupled water electrolysis using WO3and Ni(OH)2auxiliary electrodes. *Energ. Environ. Sci.* **15**, 2021–2028 (2022).

36. E. A. Toledo-Carrillo, J. Dutta, Unipolar half-cell and electrolysis system including the same. SE 2151488 (2021).

37. J. Dutta, E. A. Toledo-Carrillo, Electrolysis system including at least one capacitive half-cell. PCT/EP2022/084778 (2022).

38. M. Guo, J. Zhan, Z. Wang, X. Wang, Z. Dai, T. Wang, Supercapacitors as redox mediators for decoupled water splitting. *Chin. Chem. Lett.* **34**, 107709 (2023).

39. B. Hsia, M. S. Kim, C. Carraro, R. Maboudian, Cycling characteristics of high energy density, electrochemically activated porous-carbon supercapacitor electrodes in aqueous electrolytes. *J. Mater. Chem. A* **1**, 10518–10523 (2013).

40. S. Li, E. Li, X. An, X. Hao, Z. Jiang, G. Guan, Transition metal-based catalysts for electrochemical water splitting at high current density: Current status and perspectives. *Nanoscale* **13**, 12788–12817 (2021).

41. W. Mai, Q. Cui, Z. Zhang, K. Zhang, G. Li, L. Tian, W. Hu, CoMoP/NiFe-layered double-hydroxide hierarchical nanosheet arrays standing on ni foam for efficient overall water splitting. *ACS Appl. Energy Mater.* **3**, 8075–8085 (2020).

42. X. Wang, C. Wang, F. Lai, H. Sun, N. Yu, B. Geng, Self-supported CoFe-P nanosheets as a bifunctional catalyst for overall water splitting. *ACS Appl. Nano Mater.* **4**, 12083–12090 (2021).

43. F. Bao, E. Kemppainen, I. Dorbandt, R. Bors, F. Xi, R. Schlatmann, R. van de Krol, S. Calnan, Understanding the hydrogen evolution reaction kinetics of electrodeposited nickel-molybdenum in acidic, near-neutral, and alkaline conditions. *ChemElectroChem* **8**, 195–208 (2021).

44. W. Sheng, M. Myint, J. G. Chen, Y. Yan, Correlating the hydrogen evolution reaction activity in alkaline electrolytes with the hydrogen binding energy on monometallic surfaces. *Energ. Environ. Sci.* **6**, 1509–1512 (2013).

45. M. Liu, J. Li, Cobalt phosphide hollow polyhedron as efficient bifunctional electrocatalysts for the evolution reaction of hydrogen and oxygen. *ACS Appl. Mater. Interfaces* **8**, 2158–2165 (2016).

46. J. Kibsgaard, C. Tsai, K. Chan, J. D. Benck, J. K. Nørskov, F. Abild-Pedersen, T. F. Jaramillo, Designing an improved transition metal phosphide catalyst for hydrogen evolution using experimental and theoretical trends. *Energ. Environ. Sci.* **8**, 3022–3029 (2015).

47. Y. Chu, D. Wang, X. Shan, C. Liu, W. Wang, N. Mitsuzaki, Z. Chen, Activity engineering to transition metal phosphides as bifunctional electrocatalysts for efficient water-splitting. *Int. J. Hydrogen Energy* **47**, 38983–39000 (2022).

48. J. Yu, F. A. Garcés-Pineda, J. González-Cobos, M. Peña-Díaz, C. Rogero, S. Giménez, M. C. Spadaro, J. Arbiol, S. Barja, J. R. Galán-Mascarós, Sustainable oxygen evolution electrocatalysis in aqueous 1 M H_2SO_4 with earth abundant nanostructured Co_3O_4. *Nat. Commun.* **13**, 4341 (2022).

49. B. You, N. Jiang, M. Sheng, S. Gul, J. Yano, Y. Sun, High-performance overall water splitting electrocatalysts derived from cobalt-based metal–organic frameworks. *Chem. Mater.* **27**, 7636–7642 (2015).

50. B. H. R. Suryanto, Y. Wang, R. K. Hocking, W. Adamson, C. Zhao, Overall electrochemical splitting of water at the heterogeneous interface of nickel and iron oxide. *Nat. Commun.* **10**, 5599 (2019).

51. M. Vanags, M. Iesalnieks, L. Jēkabsons, A. Zukuls, A. Šutka, Two-step decoupled electrolysis approach based on pseudocapacitive WO3 auxiliary electrode. *Int. J. Hydrogen Energy* **48**, 20551–20561 (2023).

52. J. Kim, J. Kim, H. Kim, S. H. Ahn, Nanoporous nickel phosphide cathode for a high-performance proton exchange membrane water electrolyzer. *ACS Appl. Mater. Interfaces* **11**, 30774–30785 (2019).

53. P. Holzapfel, M. Bühler, C. Van Pham, F. Hegge, T. Böhm, D. McLaughlin, M. Breitwieser, S. Thiele, Directly coated membrane electrode assemblies for proton exchange membrane water electrolysis. *Electrochem. Commun.* **110**, 106640 (2020).

54. F. Fiévet, S. Ammar-Merah, R. Brayner, F. Chau, M. Giraud, F. Mammeri, J. Peron, J. Y. Piquemal, L. Sicard, G. Viau, The polyol process: A unique method for easy access to metal nanoparticles with tailored sizes, shapes and compositions. *Chem. Soc. Rev.* **47**, 5187–5233 (2018).

55. S. Kukunuri, M. R. Krishnan, S. Sampath, The effect of structural dimensionality on the electrocatalytic properties of the nickel selenide phase. *Phys. Chem. Chem. Phys.* **17**, 23448–23459 (2015).

56. J. Jiang, F. Sun, S. Zhou, W. Hu, H. Zhang, J. Dong, Z. Jiang, J. Zhao, J. Li, W. Yan, M. Wang, Atomic-level insight into super-efficient electrocatalytic oxygen evolution on iron and vanadium co-doped nickel (oxy)hydroxide. *Nat. Commun.* **9**, 2885 (2018).

57. B. P. Vinayan, S. Ramaprabhu, Platinum–TM (TM = Fe, Co) alloy nanoparticles dispersed nitrogen doped (reduced graphene oxide-multiwalled carbon nanotube) hybrid structure cathode electrocatalysts for high performance PEMFC applications. *Nanoscale* **5**, 5109–5118 (2013).

58. B. Vincent Crist, *Handbooks of Monochromatic XPS Spectra Volume 1 - The Elements and Native Oxides*, Handbook of The Elements and Native Oxides (XPS International, 1999).
